# Discovery and Validation of Biomarkers to Guide Clinical Management of Pneumonia in African Children

**DOI:** 10.1093/cid/ciu202

**Published:** 2014-04-02

**Authors:** Honglei Huang, Readon C. Ideh, Evelyn Gitau, Marie L. Thézénas, Muminatou Jallow, Bernard Ebruke, Osaretin Chimah, Claire Oluwalana, Henri Karanja, Grant Mackenzie, Richard A. Adegbola, Dominic Kwiatkowski, Benedikt M. Kessler, James A. Berkley, Stephen R. C. Howie, Climent Casals-Pascual

**Affiliations:** 1 Wellcome Trust Centre for Human Genetics; 2 Centre for Clinical Vaccinology and Tropical Medicine, Nuffield Department of Medicine, University of Oxford; 3 Liverpool School of Tropical Medicine, United Kingdom; 4 Child Survival Theme, Medical Research Council Unit, The Gambia;; 5 Kenya Medical Research Institute, Centre for Geographical Medicine Research (Coast), Kilifi

**Keywords:** pneumonia, malaria, lipocalin-2/NGAL, respiratory infection, biomarkers

## Abstract

***Background.*** Pneumonia is the leading cause of death in children globally. Clinical algorithms remain suboptimal for distinguishing severe pneumonia from other causes of respiratory distress such as malaria or distinguishing bacterial pneumonia and pneumonia from others causes, such as viruses. Molecular tools could improve diagnosis and management.

***Methods.*** We conducted a mass spectrometry–based proteomic study to identify and validate markers of severity in 390 Gambian children with pneumonia (n = 204) and age-, sex-, and neighborhood-matched controls (n = 186). Independent validation was conducted in 293 Kenyan children with respiratory distress (238 with pneumonia, 41 with *Plasmodium falciparum* malaria, and 14 with both). Predictive value was estimated by the area under the receiver operating characteristic curve (AUC).

***Results.*** Lipocalin 2 (Lpc-2) was the best protein biomarker of severe pneumonia (AUC, 0.71 [95% confidence interval, .64–.79]) and highly predictive of bacteremia (78% [64%–92%]), pneumococcal bacteremia (84% [71%–98%]), and “probable bacterial etiology” (91% [84%–98%]). These results were validated in Kenyan children with severe malaria and respiratory distress who also met the World Health Organization definition of pneumonia. The combination of Lpc-2 and haptoglobin distinguished bacterial versus malaria origin of respiratory distress with high sensitivity and specificity in Gambian children (AUC, 99% [95% confidence interval, 99%–100%]) and Kenyan children (82% [74%–91%]).

***Conclusions.*** Lpc-2 and haptoglobin can help discriminate the etiology of clinically defined pneumonia and could be used to improve clinical management. These biomarkers should be further evaluated in prospective clinical studies.

## Abstract (precis)

Lipocalin 2 distinguishes severe and bacterial pneumonia from nonsevere and nonbacterial pneumonia with a high level of precision. The clinical impact of this biomarker requires large-scale clinical evaluation.

Pneumonia is the leading cause of death in young children globally, accounting for almost 2 million deaths every year, mainly in developing countries [1–4]. In The Gambia, acute lower respiratory tract infection, principally pneumonia, is a leading cause of death in young children [5, 6].

To reach and push beyond the United Nations' Millennium Development Goal 4 for child survival, the number of deaths caused by pneumonia must be reduced. This will require a combination of effective preventive measures and improved clinical management [7–10]. Previous studies have shown that delayed referral is one of the most important risk factors for death in children with pneumonia [11]. Therefore, current clinical algorithms to refer patients with pneumonia to hospital are commonly based on diagnostic sensitivity rather than specificity. Consequently, overreferral of pneumonia cases has been a significant problem with the World Health Organization (WHO) case management strategy in some settings [12]. Importantly, clinical criteria do not distinguish bacterial causes of pneumonia from other causes such as viruses that do not require antibiotic treatment.

The analysis of biofluids (eg, plasma) using mass spectrometry–based methods has been widely adopted for biomarker discovery [13–15]. Once identified and validated, proteins of clinical value could be incorporated into rapid, point-of-care tests.

In African children, pneumonia is not the only cause of respiratory distress in children. In malaria endemic areas, *Plasmodium falciparum* infection may cause respiratory distress and the overlap of these conditions frequently compromises the diagnosis and management of these patients [16–18]. In acute pediatric admissions, the clinical syndrome of severe pneumonia overlapped in 39% of severe malaria cases [19].

We conducted a study to describe the plasma proteomic signature in samples from Gambian children with severe pneumonia and nonsevere pneumonia and controls. We identified and validated biomarkers to (1) predict disease severity in children with pneumonia, (2) predict blood culture positivity, (3) predict probable bacterial etiology of children with pneumonia, (4) evaluate biomarker performance to discriminate respiratory distress caused by pneumonia or by severe malaria, and (5) provide an independent validation for the diagnostic performance of these markers in Kenyan children.

## METHODS

### Study Sites and Populations Studied

The study participants were infants and children from the Greater Banjul and Basse areas aged 2–59 months taking part in a case-control study of childhood pneumonia and children admitted at the Kilifi District Hospital with a diagnosis of lower respiratory tract infection (Table 1). The sites, populations studied, and clinical definitions are described in detail in the Supplementary Methods and Supplementary Table 1.

**Table 1.** Demographic and Clinical Characteristics of the Study Participants by Outcome Group

Characteristic	Pneumonia Severity				
	Control (n = 186)	Nonsevere (n = 96)	Severe (n = 76)	Very Severe (n = 32)	*P* Value^a^
Age, median (IQR), mo	15 (8–24)	15 (8–24)	15 (8–24)	12 (6–17)	.10
Male sex, %	54.4	54.7	54.0	50.9	.95
Weight-for-age *z* score, mean (95% CI)	−0.93 (−1.01 to −0.86)	−1.26 (−1.38 to −1.13)	−1.46 (−1.59 to −1.32)	−1.41 (−1.79 to −1.03)	<.001
Respiratory rate, mean (SD), respirations/min	34 (6.66)	57 (10.1)	64 (12.1)	69 (14.0)	<.001
Oxygen saturation, mean (SD), %	98.3 (1.59)	96.5 (1.81)	95.2 (3.09)	84.6 (7.56)	<.001
Positive bacterial culture, No. (%)	…	6 (6.25)	3 (3.95)	2 (6.25)	.17

Abbreviations: CI, confidence interval; IQR, interquartile range; SD, standard deviation.

^a^ *P* values were obtained using Kruskal-Wallis or χ^2^ test for quantitative or discrete variables, respectively.

### Biomarker Discovery and Validation Studies

Plasma samples from Gambian infants and children aged 2–59 months were used for mass spectrometry–based proteomic studies (see Supplementary Methods for details). The concentrations of selected proteins (C-reactive protein [CRP], von Willebrand factor [vWF], lipocalin 2 [Lp-2] and haptoglobin) were measured with enzyme-linked immunosorbent assay (R&D Systems), according to the manufacturer's instructions.

### Data Management and Statistical Analyses

Clinical data were collected on standardized forms and double entered. Univariate and multiple logistic regression models were fitted for all clinical variables using disease severity and probable bacterial etiology as dependent variables. The interaction of independent variables was checked using the likelihood ratio test. Data were analyzed with Stata 11 software (StataCorp).

### Diagnostic Performance and Selection of Clinical Variables for Multivariate Models

The area under the receiver operating characteristic curve (AUC) was used to compare the sensitivity and specificity of selected markers. Cutoff values were chosen based on highest sensitivity and specificity to predict outcome using the roctab/detail function (Stata 11.0). When the diagnostic performance was assessed for >1 variable, the estimates were derived from a logistic regression model using the selected markers or clinical features as independent variables and the condition to diagnose as the dependent variable. These analyses were carried out using the lroc, lstat, and roctab/graph functions in Stata. The positive and negative likelihood ratios have been calculated for the biomarker concentrations with the highest sensitivity and specificity for predicting severe pneumonia.

Clinical variables and/or molecular markers were included in the multivariate models if (1) the variable showed a statistically significant association (*P* < .05) in the univariate model and (2) the variable had not been used as a criterion for a priori classification. For example, respiratory rate was combined with molecular markers to compare nonsevere and severe pneumonia because this variable was not used as a criterion to separate the 2 conditions. Ordinal univariate logistic regression models using disease severity coded as 0–3 (from control [0] to very severe [3]) as the dependent variable were used to evaluate the significance of the association between biomarker concentration and clinical outcome.

### Ethical Approval

Written informed consent was given by the parent or guardian of each participant. Joint Gambia Government/Medical Research Council (MRC) Ethical Committee approval was obtained for both the pneumonia study (SCC/EC 1062) and the severe malaria study (SCC/EC 630 and 670). The use of the archived plasma samples from Kenya was approved by the Kenya Medical Research Institute (KEMRI) Ethics Review Committee (SSC 2280).

## RESULTS

### Diagnostic Performance of Clinical Features and Protein Biomarkers to Discriminate Severe Pneumonia and Nonsevere Pneumonia

The proteomic analysis identified 238 proteins in children with severe pneumonia, 316 in children with nonsevere pneumonia, and 268 in healthy controls. The difference in protein numbers across different groups and batches was not significantly different (analysis of variance, *P* = .35). We identified 23 differentially regulated proteins (>1.5 fold) that were present in ≥2 of 3 batches when severe and nonsevere cases were compared and 19 when nonsevere cases and controls were compared (Supplementary Figure 2). Of these 42 proteins, only 8 (19%) indicated a progression pattern, namely, increasing concentration from controls to mild to severe cases. Based on the number of peptides used to identify these proteins and their biological relevance, Lpc-2, CRP, and vWF were selected for further validation (Table 2).

**Table 2.** Molecular Marker Concentrations by Outcome Group

Molecular Marker	Pneumonia Severity				
	Control	Mild	Severe	Very Severe	*P* Value^a^
Median concentration (IQR) [No.]					
Lpc-2, ng/mL	59.8 (44.3–78.4) [174]	92.0 (51.6–144) [77]	152 (87.6–283) [73]	224 (139–455) [32]	<.001
Haptoglobin, mg/mL	1.22 (0.8–1.94) [160]	1.9 (1.2–2.8) [79]	2.6 (1.8–4.4) [76]	3.8 (2.5–4.9) [32]	<.001
vWF, mU/mL^b^	731 (459–1082) [96]	757 (480–1361) [67]	1260 (751–1725) [68]	1955 (1337–2459) [32]	<.001
CRP, µg/mL^b^	6.19 (4.78–20.1) [55]	93.1 (17.9–227) [77]	258 (184–332) [65]	116 (63–230) [24]	<.001

Abbreviations: CRP, C-reactive protein; IQR, interquartile range; Lpc-2, lipocalin 2; vWF, von Willebrand factor.

^a^ *P* values are derived from an ordinal univariate logistic regression model wherein the dependent variable (disease severity) is coded as 0–3 (from control [0] to very severe [3]) and the independent variables are the concentrations of the biomarkers. Differences in the number of samples tested for different biomarkers depended on sample availability and the number of tests required to measure concentration within linear range of the enzyme-linked immunosorbent assay standard.

^b^ The sample volume available for enzyme immunoassay measurements was limited, and Lpc-2 and haptoglobin were prioritized over vWF or CRP. This accounts for the discrepancies observed in the number of samples tested for CRP and vWF.

**Figure 1.**
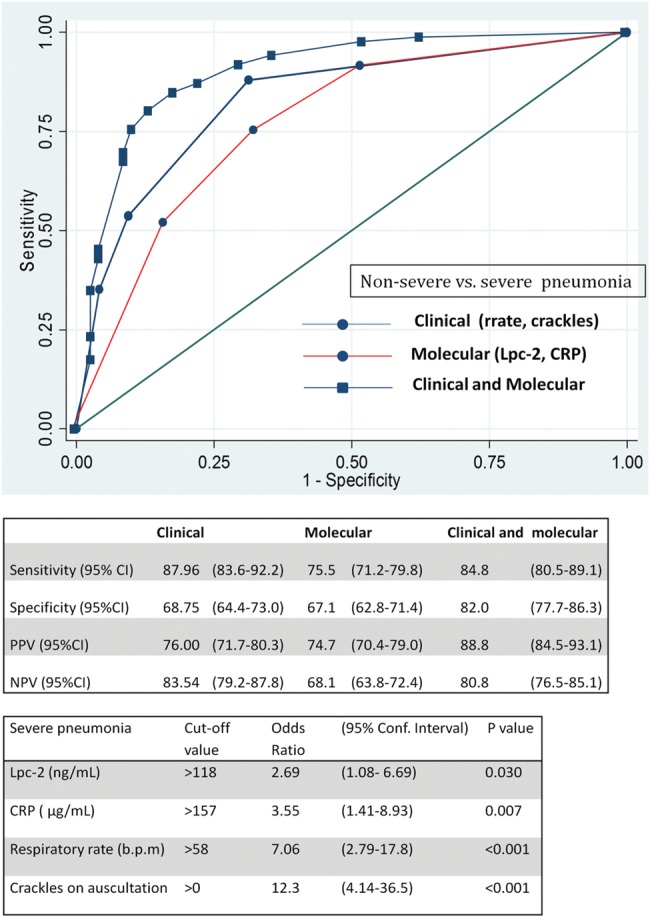
Diagnostic performance of clinical and molecular markers to predict severe pneumonia (vs nonsevere pneumonia) in Gambian children. Only clinical features that were not used to distinguish the 2 groups were introduced in the model (see Methods). Odds ratios (with 95% confidence intervals [CIs]) indicate odds of severe pneumonia for cutoff values with the highest sensitivity and specificity. Abbreviations: CRP, C-reactive protein; Lpc-2, lipocalin 2; NPV, negative predictive value; PPV, positive predictive value.

Lpc-2, CRP, and vWF levels were significantly higher in children with severe pneumonia than in those with nonsevere pneumonia (Supplementary Figure 3). Lpc-2 was the best predictor of severe pneumonia, with a sensitivity of 72.3% and a specificity of 70.1% (AUC, 0.71 [95% confidence interval [CI], .64–.79]; Table 3). In children with Lpc-2 levels >118 ng/mL, the odds of having severe disease increased by nearly 3-fold (odds ratio [OR], 2.69 [95% CI, 1.08–6.69). A CRP concentration >157 µg/mL was associated with increased disease severity (OR, 3.55 [95% CI, 1.41–8.93]), but despite its good sensitivity to predict disease severity (70.8%), its specificity was low (56.2%). Similarly, plasma vWF concentrations >648 mU/mL were associated with a 5-fold increase in the odds of severe pneumonia (OR, 5.26 [95% CI, 2.42–11.4]), with good sensitivity (87.0%) but poor specificity (41.7%; Figure 1).

**Table 3.** Diagnostic Performance of Clinical Features and Molecular Markers Associated With a Risk of Severe or Very Severe Pneumonia (vs Nonsevere Pneumonia)

			Likelihood Ratio^a^	
Clinical Feature	Children, No.	AUC (95% CI)	Positive	Negative
Respiratory rate^b^	204	0.76 (.70–.83)	…	…
Crackles^b^	204	0.72 (.66–.77)	…	…
Heart rate	204	0.68 (.61–.75)	…	…
Pallor	204	0.57 (.52–.61)	…	…
Molecular marker				
Lpc-2^b^	182	0.71 (.64–.79)	2.39	0.40
CRP^b^	166	0.68 (.60–.76)	1.66	0.53
vWF^b^	167	0.70 (.62–.78)	2.16	0.46

Abbreviations: AUC, area under the receiver operating characteristic curve; CI, confidence interval; CRP, C-reactive protein; Lpc-2, lipocalin 2; vWF, von Willebrand factor.

^a^ The positive and negative likelihood ratios have been calculated for the biomarker concentrations with the highest sensitivity and specificity for predicting severe pneumonia (Lpc-2, 118 ng/mL; CRP, 157 mg/mL; and vWF, 648 mU/mL).

^b^ Variables independently associated with outcome in the multivariate models.

The best combination of clinical and molecular markers to predict severe pneumonia included respiratory rate, crackles, Lpc-2, and CRP. The addition of molecular markers to the clinical features had no impact on sensitivity but increased specificity from 68.0% to 82.0% (Figure 1), and the effect was superior to radiological changes (end point consolidation), crackles, and positive blood culture combined (Supplementary Figure 6). The sensitivity of this combination of markers increased to 94.7% (95% CI, 88.4%–100%) in children enrolled during the dry season, possibly owing to the absence of malaria cases (Supplementary Figure 4). Only 3 children of 204 (1.5%) identified as having pneumonia had a positive malaria slide. These children all had nonsevere pneumonia (2 enrolled during the dry season and 1 during the rainy season).

### Association of Lpc-2 With Probable Bacterial Pneumonia

**Figure 2.**
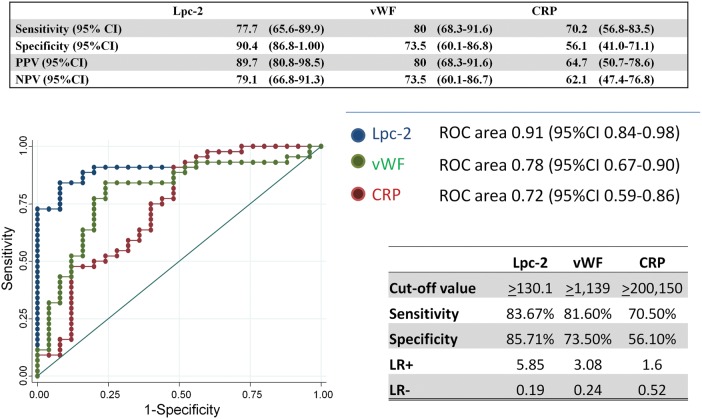
Diagnostic performance of lipocalin 2 (Lpc-2), von Willebrand factor (vWF) and C-reactive protein (CRP) in predicting probable bacterial infection. Abbreviations: AUC, area under the receiver operating characteristic curve; CI, confidence interval; LR, likelihood ratio; NPV, negative predictive value; PPV, positive predictive value; ROC, receiving operating characteristic.

Bacterial cultures were performed on 198 of 200 patients with pneumonia (99%). Of these, 12 of 198 (6%) were positive for true pathogens, namely *Streptococcus pneumoniae* (n = 10), *Staphylococcus aureus* (n = 1), and *Streptococcus pyogenes* (n = 1). The contamination rate was 6%. None of the clinical variables were associated with a positive blood culture (Supplementary Table 6), but children with a concentration of Lpc-2 >163 ng/mL had 9 times the odds of having a positive blood culture with a clinically significant isolate (OR, 9.03 [95% CI, 1.91–42.6]; Supplementary Figure 11). The plasma concentrations of Lpc-2, CRP, and vWF in children with a positive blood culture or with “end point” consolidation on their chest radiograph (probable bacterial pneumonia) were significantly higher than in children with no consolidation and a low white blood cell count (*P* < .01; Table 4). Lpc-2 concentrations were strongly associated with pneumonia of probable bacterial origin (Figure 2). The diagnostic performance of Lpc-2 was superior to that of vWF or CRP. Lpc-2 showed good sensitivity (77% [95% CI, 65.6%–89.9%]) and very high specificity (94.4% [95% CI, 86.8%–100%]) for identifying children with probable bacterial pneumonia. Similar results were obtained when patients with a positive blood culture were excluded from the analysis (OR, 22.5 [95% CI, 6.77–74.7]).

**Table 4.** Population Description of Patients With Pneumonia of Probable Bacterial or Probable Viral Etiology

Characteristic	Pneumonia		
	Probable Non-bacterial (n = 49)	Probable Bacterial (n = 54)	*P* Value^a^
Age, mean (SD), mo	19 (10.5)	17.4 (11.6)	.14
Male sex, %	50%	51%	.90
Weight-for-age *z* score, mean (SD)	−1.03 (1.10)	−1.37 (1.21)	.24
Respiratory rate, mean (SD), respirations/min	57.0 (12.0)	62.6 (9.8)	.007
Oxygen saturation, mean (SD), %	96.3 (2.64)	92.84 (7.09)	<.01
WBC count, mean (SD), ×10^9^/L	10.1 (6.42)	23.7 (10.6)	<.01
Hemoglobin, mean (SD), g/dL	10.3 (1.53)	8.65 (1.74)	<.01
Molecular marker concentration, median (IQR) [No.]			
Lpc-2, ng/mL	[42] 81.7 (45.7–109)	[45] 282 (155–365)	<.001
CRP, µg/mL	[41] 175 (182–278)	[47] 283 (142–350)	<.001
vWF, mU/mL	[34] 810 (647–1201)	[45] 1514 (1205–2036)	<.001

Abbreviations: CRP, C-reactive protein; IQR, interquartile range; Lpc-2, lipocalin 2; SD, standard deviation; vWF, von Willebrand factor; WBC, white blood cell.

^a^ *P* values were obtained using Mann-Whitney or χ^2^ test for quantitative or discrete variables, respectively.

### Haptoglobin in Discriminating Between Pneumonia and Malaria-Associated Respiratory Distress in Gambian Children

**Figure 3.**
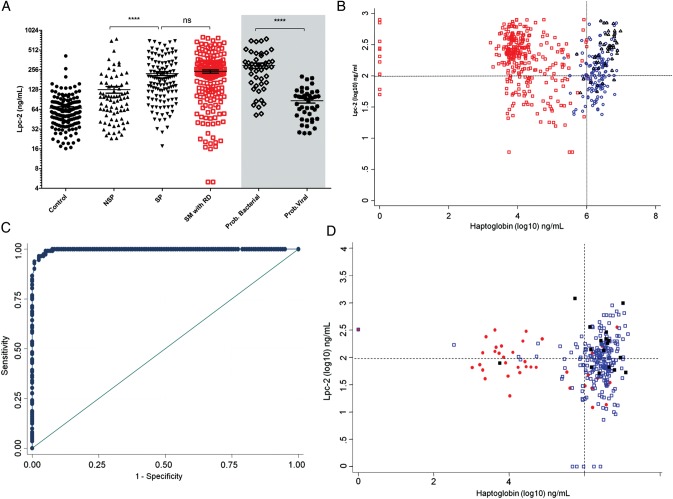
Discrimination between pneumonia and malaria-associated respiratory distress in Gambian children. *A*, Plasma concentration of lipocalin 2 (Lpc-2) in children with different syndromes of respiratory distress and controls. Gray shaded area indicates subanalysis of cases defined as probable bacterial or probable nonbacterial pneumonia. *B,* Distribution of cases of respiratory distress based on plasma levels of Lpc-2 and haptoglobin. *C,* Diagnostic performance (receiving operating characteristic curve) of haptoglobin in discriminating between respiratory distress caused by pneumonia and severe malaria with respiratory distress. *D,* Validation of diagnostic performance in an independent population of Kenyan children. *P < .0001; Abbreviations: NS, nonsignificant; NSP, nonsevere pneumonia; RD, respiratory distress; SM, severe malaria; SP, severe pneumonia.

Lpc-2 concentrations in children with pneumonia did not differ significantly from those in children with severe malaria and respiratory distress (Figure 3*A*). We reasoned that a marker of hemolysis such as haptoglobin could discriminate respiratory distress caused by pneumonia from that caused by severe malaria because haptoglobin levels increase with pneumonia severity and decrease with malaria severity owing to erythrocyte destruction. The median (interquartile range) plasma haptoglobin concentration in children with severe malaria and respiratory distress (10 406 [5267–38 597] ng/mL) was 2 orders of magnitude lower than that in children with severe pneumonia (2 618 150 [1 800 000–4 400 000] ng/mL [*P* < .001]; Supplementary Figure 7). The sensitivity and specificity of haptoglobin (>1.1 mg/mL) in discriminating respiratory distress caused by pneumonia from that caused by malaria were 92.8% and 99.2%, respectively (Figure 3*B*). Children with probable bacterial pneumonia presented with the highest concentrations of haptoglobin and Lpc-2 (Supplementary Figure 3*C*). The combination of Lpc-2 and haptoglobin were effective in discriminating between pneumonia and severe malaria with respiratory distress (Figure 3*C* and 3*D*). Only 2 of 307 patients (0.65%) presented with the hemoglobin S mutation of the β-globin chain (SS genotype). The haptoglobin concentrations did not differ significantly between patients with AS or SS genotype and those with AA phenotype.

### Lpc-2 and Haptoglobin in Discriminating Between Pneumonia and Malaria-Associated Respiratory Distress in Kenyan Children

The diagnostic performance of Lpc-2 and haptoglobin in discriminating respiratory distress caused by pneumonia or malaria was evaluated in an independent population of 293 Kenyan children (Table 5). The same cutoff values derived from the Gambian data set were applied (Figure 3*D*). In children admitted to the hospital who had a plasma haptoglobin concentration >1.1 mg/mL, respiratory distress was 14.8 times more likely to be caused by pneumonia than by malaria (OR, 14.8 [95% CI, 7.05–31.2]) . The diagnostic performance based on sensitivity and specificity was high but expectedly lower than that observed in Gambian children (AUC, 0.82 [95% CI, .73–.91]). The proportion of children with severe malaria and a high haptoglobin concentration (11 of 41 [27%]) was the major difference observed between the Kenyan and the Gambian study. We observed a bimodal distribution in haptoglobin concentrations (above and below 5 × 10^6^ ng/mL) in children with malaria and respiratory distress and in children with both pneumonia and malaria (Supplementary Figure 8).

**Table 5.** Population Description of Kenyan Patients With Respiratory Distress Caused by Malaria, Pneumonia, or Both

Characteristic	Malaria (n = 41)	Pneumonia (n = 238)	Pneumonia and Malaria (n = 14)	*P* Value^a^
Age, median (IQR), mo	21.1 (9.3–33.2)	12.8 (7.2–34.5)	30.4 (16.9–51.9)	.03
Male sex, %	53.6	54.6	35.7	.38
Weight-for-age *z* score, mean (SD)	−1.73 (1.48)	−2.06 (1.45)	−2.07 (1.43)	.48
Respiratory rate, median (IQR), respirations/min	56.4 (12.1)	56 (46–64)	52 (42–58)	.29
Oxygen saturation, mean (SD), %	99 (96–100)	96 (92–98)	97 (96–99)	<.01
WBC count, median (IQR), ×10^9^/L	11.4 (7.9–17.1)	14 (9.9–20.9)	10.5 (6.7–21.5)	.08
Hemoglobin, mean (SD), g/dL	7.35 (4.8–9)	9 (7.9–9.9)	8.1 (5.3–8.7)	<.01
Positive bacterial culture, No. (%)^b^	2 (4.88)	16 (6.72)	0 (0)	<.01
Lpc-2, median (IQR), ng/mL	72.2 (44–151)	92.8 (51.1–159.6)	195 (51.3–286)	.46
Haptoglobin, median (IQR), ng/mL	23 434 (5445–1 048 424)	3 095 352 (1 770 148–4 383 530)	19 106 (8353–1 250 607)	<.001

Abbreviations: IQR, interquartile range; Lpc-2, lipocalin 2; SD, standard deviation; WBC, white blood cell.

^a^ *P* values were obtained using Kruskal-Wallis or χ^2^ for quantitative or discrete variables, respectively.

^b^ Positive bacterial culture include the following significant microorganisms: *Streptococcus pneumoniae* (13 samples), *Salmonella* spp. (2 samples), and *Staphylococcus aureus*, *Escherichia coli,* and β-hemolytic streptococcus (1 sample each).

The odds of a positive bacterial culture with a significant bacterial isolate were significantly higher in children with higher plasma concentrations of Lpc-2 (OR, 5.62 [95% CI, 1.60–19.6]). However, the diagnostic performance of Lpc-2 in predicting bacteremia was lower than in Gambian children (AUC, 0.67 [95% CI, .54–.79]). In Kenyan children, nutritional status, measured with the height-for-age *z* score and weight for age, was inversely correlated with Lpc-2 concentration (r = −0.13 [*P* = .02] and r = −0.12 [*P* = .03]). These differences were not observed in Gambian children. We therefore reasoned that differences in the proportion of malnourished children in the Kenyan and Gambian populations may account for differences in the diagnostic performance of Lpc-2. Indeed, the proportion of children with stunting (measured with the height-for-age *z* score) was higher in Kenyan children than in Gambian children. The height-for-age *z* score (but not other measures of nutritional status) and the presence of oral candidiasis were independently associated with bacteremia. The diagnostic performance of Lpc-2 in predicting blood stream infection increased to an AUC of 78.7% (95% CI, 60%–86%) after adjustment for these 2 variables (Supplementary Figure 10). The human immunodeficiency virus (HIV) status was available for 163 of 293 patients (55.6%) and was positive in 30 patients (18.4%). Of 12 patients with oral candidiasis, 7 were HIV negative (58.3%). HIV was not associated with blood stream infection in the population studied (OR, 1.53 [95% CI, .38–6.03]). The diagnostic performance of Lpc-2 in predicting bacteremia was higher in younger children (aged <13.9 months) after adjustment for stunting (AUC, 86%), whereas haptoglobin performed better in older children (>13.9 months) regardless of nutritional status (Supplementary Figure 9).

## DISCUSSION

This study shows that Lpc-2 is a biomarker associated with severe pneumonia, specifically with blood culture positivity and pneumonia designated as being of probable bacterial origin based on radiological and other criteria. Furthermore, the combination of Lpc-2 with haptoglobin discriminates between pneumonia and malaria-associated respiratory distress.

To reduce the number of deaths caused by pneumonia, early diagnosis is critical for pneumonia cases due to bacterial infection or likely to become severe, so appropriate treatment can be administered promptly. However, the diagnosis of bacterial pneumonia is usually compromised by the lack of specificity of respiratory symptoms, which are commonly shared with other conditions that cause respiratory distress in children, many associated with high case fatality rates. In sub-Saharan Africa *P. falciparum* malaria and bacterial blood stream infections are frequent causes of respiratory distress in children and possibly pathogenically linked [20]. For a biomarker to be helpful in this clinical context, its diagnostic performance has to be superior to clinical examination, and its specificity must be sufficient to guide clinical management.

The WHO definition of pneumonia severity used in our study is an operational clinical algorithm rather than a “gold standard.” This definition aims to reduce mortality by improving referral practices and thus prioritizes sensitivity over specificity. The WHO has recently proposed a more specific definition [21]. The starting hypothesis of our study was that the addition of molecular markers could increase the specificity of the clinical definition of severe pneumonia. In this context, we have reported 3 potential plasma biomarkers that alone or in association with clinical features could be used to improve clinical management by identifying pneumonia with high disease severity or probable bacterial etiology.

Our results indicate that CRP and Lpc-2 can correctly distinguish most patients with severe pneumonia from those with nonsevere pneumonia and probable bacterial from probable nonbacterial causes. The sensitivity of these markers combined increased to 94.7% when the analysis was performed exclusively in children with pneumonia recruited during the dry season. The specificity of this panel of markers decreased from 82% to 77% when the analysis was performed in children enrolled during the rainy season (Supplementary Figure 4). Owing to the high seasonality of malaria transmission in The Gambia and the fact that both CRP and Lpc-2 have been shown to increase in children with malaria [22, 23], it is plausible that this increase in sensitivity and specificity is explained by the low number of malaria infections during the dry season. This increase in diagnostic specificity is likely to reduce but not completely remove the number of false-positive cases burdening busy health services. Similarly, our panel of biomarkers performed well against the more recent definition for severity proposed by the WHO in 2013 (Supplementary Figure 5).

Here we report that Lpc-2 is associated with blood culture positivity. This is particularly important because clinical features did not identify children with a positive bacterial blood culture. We did not confirm findings from some previous studies reporting an association between bacterial infection and the clinical signs of respiratory distress or high temperature (Supplementary Table 6) [24–28]. To our knowledge, we are also the first to report that Lpc-2 is associated with pneumonia of probable bacterial origin, defined by the presence of consolidation on the chest radiograph or a positive blood culture. This was true for both primary bacterial and nonbacterial pneumonia definitions. The association of Lpc-2 with positive blood cultures and pneumonia of probable bacterial origin is biologically plausible and clinically important. We also observed that the association of Lpc-2 and bacteremia in Kenyan children, although significant, was partially compromised by the patient's nutritional status. This observation may be explained, at least in part, by the reduced ability of patients with chronic malnutrition to generate an effective immune response to infection. This limitation may be important in the design of prospective studies, and different cutoff values may be required for populations with severe stunting.

The role of Lpc-2 in innate defense against bacterial infection is well established. The transcription of the Lpc-2 gene has been shown to be up-regulated in activated macrophages through Toll-like receptor 4 ligation and to interfere with bacterial iron uptake [29, 30]. More importantly, Lpc-2 transcription is increased by 65-fold in the nasal mucosa of mice in response to *S. pneumoniae* and *Haemophilus influenzae* colonization [31].

In resource-limited settings where laboratory facilities and radiology are rarely available to help diagnose bacterial pneumonia cases, molecular markers such as Lpc-2 could be developed into a point-of-care diagnostic tool to target cases that require antibiotic treatment. Indeed, Lpc-2 concentrations could be used to stop antibiotic treatment in patients who show a quick clinical recovery. Similarly, for acute respiratory infections at the community level, Lpc-2 could be used to determine the indications for and guide the timing of antibiotic treatment [32–35]. The proteomic workflow used in this study could not identify proteins at concentrations below the mid–nanogram-per-milliliter range [14], such as tumor necrosis factor, interleukin 6, and procalcitonin, previously shown to be associated with blood stream infection [36].

We found that haptoglobin in combination with Lpc-2 was remarkably effective in discriminating between pneumonia and malaria in children with respiratory distress. Two major reasons account for this observation. Firstly, hemolysis is a major feature of acute malaria infection but not pneumonia [37]. During active hemolysis, free hemoglobin binds to haptoglobin, leading to a rapid depletion of this molecule from plasma [38]. Secondly, the concentration of plasma haptoglobin increased with disease severity in patients with Pneumonia.

Point-of-care diagnostics can help in making rapid clinical decisions in developing countries. Clinical efficacy and cost-effectiveness analysis studies are required to estimate the potential impact of these novel tools [39]. In malaria-endemic areas, the combination of Lpc-2 with haptoglobin should be prospectively evaluated for use in guiding the clinical management of children with respiratory distress.

## Supplementary Data

Supplementary materials are available at *Clinical Infectious Diseases* online (http://cid.oxfordjournals.org). Supplementary materials consist of data provided by the author that are published to benefit the reader. The posted materials are not copyedited. The contents of all supplementary data are the sole responsibility of the authors. Questions or messages regarding errors should be addressed to the author.

Supplementary Data

## References

[1] Black RE, Morris SS, Bryce J (2003). Where and why are 10 million children dying every year?. Lancet.

[2] Mathers CD, Claudia S, Fat DM Global Burden of Disease 2000: version 2 methods and results. http://www.who.int/healthinfo/paper50.pdf.

[3] Mulholland K (2003). Global burden of acute respiratory infections in children: implications for interventions. Pediatr Pulmonol.

[4] United Nations (2001). Road map towards the implementation of the United Nations Millennium Declaration: report of the Secretary General. 56th session of the United Nations General Assembly. http://www.un.org/millenniumgoals/sgreport2001.pdf.

[5] Greenwood BM, Greenwood AM, Bradley AK, Tulloch S, Hayes R, Oldfield FS (1987). Deaths in infancy and early childhood in a well-vaccinated, rural, West African population. Ann Trop Paediatr.

[6] Jaffar S, Leach A, Greenwood AM (1997). Changes in the pattern of infant and childhood mortality in upper river division, The Gambia, from 1989 to 1993. Trop Med Int Health.

[7] Howie SR, Adegbola RA (2006). Pneumonia and child mortality. Lancet.

[8] Madhi SA, Levine OS, Hajjeh R, Mansoor OD, Cherian T (2008). Vaccines to prevent pneumonia and improve child survival. Bull World Health Organ.

[9] Niessen LW, ten Hove A, Hilderink H, Weber M, Mulholland K, Ezzati M (2009). Comparative impact assessment of child pneumonia interventions. Bull World Health Organ.

[10] Scott JA (2008). The global epidemiology of childhood pneumonia 20 years on. Bull World Health Organ.

[11] Reyes H, Perez-Cuevas R, Salmeron J, Tome P, Guiscafre H, Gutierrez G (1997). Infant mortality due to acute respiratory infections: the influence of primary care processes. Health Policy Plan.

[12] Bari A, Sadruddin S, Khan A (2011). Community case management of severe pneumonia with oral amoxicillin in children aged 2–59 months in Haripur district, Pakistan: a cluster randomised trial. Lancet.

[13] Aebersold R, Mann M (2003). Mass spectrometry-based proteomics. Nature.

[14] Anderson NL, Anderson NG (2002). The human plasma proteome: history, character, and diagnostic prospects. Mol Cell Proteomics.

[15] Nesvizhskii AI, Aebersold R (2005). Interpretation of shotgun proteomic data: the protein inference problem. Mol Cell Proteomics.

[16] Berkley J, Mwarumba S, Bramham K, Lowe B, Marsh K (1999). Bacteraemia complicating severe malaria in children. Trans R Soc Trop Med Hyg.

[17] Berkley JA, Maitland K, Mwangi I (2005). Use of clinical syndromes to target antibiotic prescribing in seriously ill children in malaria endemic area: observational study. BMJ.

[18] English M, Punt J, Mwangi I, McHugh K, Marsh K (1996). Clinical overlap between malaria and severe pneumonia in Africa children in hospital. Trans R Soc Trop Med Hyg.

[19] Mwaniki MK, Nokes DJ, Ignas J (2009). Emergency triage assessment for hypoxaemia in neonates and young children in a Kenyan hospital: an observational study. Bull World Health Organ.

[20] Scott JA, Berkley JA, Mwangi I (2011). Relation between falciparum malaria and bacteraemia in Kenyan children: a population-based, case-control study and a longitudinal study. Lancet.

[21] World Health Organization Pocket book of hospital care for children: second edition. http://www.who.int/maternal_child_adolescent/documents/child_hospital_care/en/.

[22] Diez-Padrisa N, Bassat Q, Machevo S (2010). Procalcitonin and C-reactive protein for invasive bacterial pneumonia diagnosis among children in Mozambique, a malaria-endemic area. PLoS One.

[23] Mohammed AO, Elghazali G, Mohammed HB (2003). Human neutrophil lipocalin: a specific marker for neutrophil activation in severe *Plasmodium falciparum* malaria. Acta Trop.

[24] Banya WA, O'Dempsey TJ, McArdle T, Lloyd-Evans N, Greenwood BM (1996). Predictors for a positive blood culture in African children with pneumonia. Pediatr Infect Dis J.

[25] Zimmermann O, de Ciman R, Gross U (2005). Bacteremia among Kenyan children [letter]. N Engl J Med.

[26] Falade AG, Adegbola RA, Mulholland EK, Greenwood BM (2001). Respiratory rate as a predictor of positive lung aspirates in young Gambian children with lobar pneumonia. Ann Trop Paediatr.

[27] Lin CJ, Chen PY, Huang FL, Lee T, Chi CS, Lin CY (2006). Radiographic, clinical, and prognostic features of complicated and uncomplicated community-acquired lobar pneumonia in children. J Microbiol Immunol Infect.

[28] Moreno L, Krishnan JA, Duran P, Ferrero F (2006). Development and validation of a clinical prediction rule to distinguish bacterial from viral pneumonia in children. Pediatr Pulmonol.

[29] Bachman MA, Miller VL, Weiser JN (2009). Mucosal lipocalin 2 has pro-inflammatory and iron-sequestering effects in response to bacterial enterobactin. PLoS Pathog.

[30] Flo TH, Smith KD, Sato S (2004). Lipocalin 2 mediates an innate immune response to bacterial infection by sequestrating iron. Nature.

[31] Nelson AL, Barasch JM, Bunte RM, Weiser JN (2005). Bacterial colonization of nasal mucosa induces expression of siderocalin, an iron-sequestering component of innate immunity. Cell Microbiol.

[32] Agarwal G, Awasthi S, Kabra SK, Kaul A, Singhi S, Walter SD (2004). Three day versus five day treatment with amoxicillin for non-severe pneumonia in young children: a multicentre randomised controlled trial. BMJ.

[33] Awasthi S, Agarwal G, Kabra SK (2008). Does 3-day course of oral amoxycillin benefit children of non-severe pneumonia with wheeze: a multicentric randomised controlled trial. PLoS One.

[34] Greenberg D, Givon-Lavi N, Sadaka Y, Ben-Shimol S, Ziv JB, Dagan R (2014). Short course antibiotic treatment for community-acquired alveolar pneumonia in ambulatory children: a double blind, randomized, placebo controlled trial. Pediatr Infect Dis J.

[35] Hazir T, Nisar YB, Abbasi S (2011). Comparison of oral amoxicillin with placebo for the treatment of world health organization-defined nonsevere pneumonia in children aged 2–59 months: a multicenter, double-blind, randomized, placebo-controlled trial in Pakistan. Clin Infect Dis.

[36] Reinhart K, Bauer M, Riedemann NC, Hartog CS (2012). New approaches to sepsis: molecular diagnostics and biomarkers. Clin Microbiol Rev.

[37] Lamikanra AA, Brown D, Potocnik A, Casals-Pascual C, Langhorne J, Roberts DJ (2007). Malarial anemia: of mice and men. Blood.

[38] Nagel RL, Gibson QH (1971). The binding of hemoglobin to haptoglobin and its relation to subunit dissociation of hemoglobin. J Biol Chem.

[39] Drain PK, Hyle EP, Noubary F (2014). Diagnostic point-of-care tests in resource-limited settings. Lancet Infect Dis.

