# Supplementary material for: Discovery and Validation of Biomarkers to Guide Clinical Management of Pneumonia in African Children
Source: Clin Infect Dis. 2014 Apr 2;58(12):1707–15. doi: 10.1093/cid/ciu202 (PMC4036688; doi:10.1093/cid/ciu202)
Supplement: Supplementary Data [file supp_58_12_1707__index.html]

Discovery and Validation of Biomarkers to Guide Clinical Management of Pneumonia in African Children — Supplementary Data 

# Discovery and Validation of Biomarkers to Guide Clinical Management of Pneumonia in African Children

## Supplementary Data

Supplementary Data

**Files in this Data Supplement:**

- Supplementary Data - Pdf file
