# Supplementary material for: Discovery and Validation of Biomarkers to Guide Clinical Management of Pneumonia in African Children
Source: Clin Infect Dis. 2014 Apr 2;58(12):1707–15. doi: 10.1093/cid/ciu202 (PMC4036688; doi:10.1093/cid/ciu202)
Supplement: Supplementary Data [file supp_ciu202_ciu202supp.pdf]

Supplementary Material

Methods

Sites and population studied

Gambia

1) *Pneumonia study*. The study participants were children from the Greater Banjul and Basse areas aged 2 to 59 months taking part in a study of childhood pneumonia. Severe cases, non-severe cases and controls were selected through assignment of randomly generated numbers and selection of 100 patients per group from study participants recruited in the Greater Banjul area between 28 June 2007 and 30 June 2010. These cases were identified at five health facilities in this area (the Royal Victoria Teaching Hospital, the Medical Research Council Hospital, and the major health centers at Serekunda, Fajikunda and Brikama). Controls from the community were individually matched to cases by age, sex, neighborhood and season.
2) *Severe malaria study.* The population studied has been described elsewhere [1]. Briefly, children aged 4 months to 15 years were eligible for enrolment if they had a blood smear positive for asexual *P. falciparum* parasites and met one or more of the WHO criteria for severe malaria.[2] Chest X-ray was not performed on these children. Only children under 5 years of age were included in the analysis.

Kenya

1) *Validation study:* Archived plasma samples were drawn from children aged between 2 to 140 months recruited for studies investigating the role of the development of natural immunity to malaria and long-term surveillance of childhood LRTI admissions in Kilifi District Hospital were used in the study. Samples were randomly selected from children admitted to the hospital between June 2006 and June 2007.

Clinical definitions

Pneumonia definitions were modified from the World Health Organization (WHO) criteria [3]. Non-severe pneumonia was defined by the presence of cough or difficulty in breathing plus tachypnea (≥50/minute age 2-11 months, ≥40/minute age ≥12) and no signs of severity; severe pneumonia was defined as cough or difficulty in breathing plus respiratory distress

(lower chest wall indrawing or nasal flaring). Probable bacterial pneumonia was defined by the presence of a positive blood culture or by the presence of clear consolidation in the chest X-ray ("end-point" consolidation according to the WHO definition) and/or pleural effusion [4]. Probable non-bacterial pneumonia was defined by a normal chest X-ray and a low white blood cell count (<10,000x10^9/L) in children without "end-point" consolidation on the chest X-ray.

All cases had a chest radiograph taken at recruitment. The radiographs were assessed according to WHO criteria by a trained clinician [5]. Blood cultures were obtained from all patients with non-severe and severe disease. Blood cultures were performed using the BACTEC 9050 system (BD, New Jersey, USA) by direct inoculation of culture media using standard microbiological procedures[6]. A blood culture was considered positive if a significant bacterial isolate was grown and identified from a colony sub-cultured in solid medium. *Micrococci* spp (N=3), coagulase-negative staphylococci (N=6) and *Bacillus* spp (N=3) were considered culture contaminants. The blood volume inoculated for culture was not measured in this study. However, data from other studies using the same extraction protocol indicate that the volumes are within the recommended range, namely1.9 ml (IQR 1.4-2.3) (MRC Gambia Unit) and 1.2 mL (0.6-1.9) in the Kenyan study [7]. The prevalence of occult bacteremia in a previous study of Kenyan children presenting with **illnesses treated as outpatients was 0.5% [8].**

To evaluate the association of Lpc-2 with blood culture positivity we defined two distinct etiological groups of patients: probable bacterial and probable non-bacterial pneumonia (most likely viral), based on radiological findings and white blood cell count. Chest radiographs were performed in 166 of 204 (81.0%) children with pneumonia. Of these, 155 (93.3%) were of adequate quality and 136 (87.7%) showed significant pathology: 37 (27.2%) 'end-point' consolidation, 6 (4.40%) pleural effusion and 113 (83.1%) 'other infiltrates' as defined by the WHO definition. [5]

Respiratory distress in severe malaria patients was defined by the presence of an abnormal respiratory pattern, grunting, lower chest wall indrawing, use of accessory muscles of respiration, or abnormally deep (acidotic) breathing.

Proteomic studies

The initial biomarker discovery phase was conducted on clinical samples from the Gambian cohort. The rational to include 100 individual per group was based on 1) the sample volume required for HPLC depletion and gel separation and adequacy for mass spectrometry analysis, and 2) to ensure that the confidence interval around the estimated sensitivity/specificity is sufficiently narrow to derive meaningful conclusions. For example, a candidate biomarker with a sensitivity and specificity higher than 75%, the maximum 95% confidence interval will be 8% below and above our point estimate, namely from 67% to 83%.Samples were divided into three different groups of 100 children according to disease severity (see Supplementary Figure 1). Individual samples (5 µl of plasma) were pooled into 3 different groups (~165µl of plasma per batch) in each disease category (control, non-severe and severe pneumonia). Pooled plasma samples were depleted of the top 14 highly-abundant plasma proteins with a multiple affinity removal (MARS) column (Agilent, UK) using high-performance liquid-chromatography (HPLC) 1200 series (Agilent, UK). Proteins from depleted plasma were precipitated with trichloroacetic acid and quantified using a colorimetric assay (BCA Protein assay, Thermo Scientific, US) and further separated by size using SDS-PAGE. Protein bands (13 bands per sample) were cut and digested with trypsin. Peptide digests were purified using Sep-Pak C18 columns (Waters, Milford, MA). Samples were analysed in a nano LC-MS/MS (Agilent 1200 HPLC with a Chip cube coupled to a 6520 Q-TOF, Agilent, UK) and searched against the human proteome with a false-discovery rate of 1% calculated from target-decoy hits and relative (label-free) quantification was based on normalized spectral index quantitation (SINQ) [9, 10]. Clinically and biologically relevant proteins that were up- regulated or down-regulated following a disease progression pattern (SP>NSP>C or vice versa) in two batches or more were selected as candidate biomarkers (see supplementary Figure 1 and supplementary Table 1).

Plasma depletion| Each sample was delipidated by centrifugation at 10,000 x g for 10 min and clear plasma was collected in a new tube. The samples were diluted 8 times in buffer A (Agilent technologies, UK) and filtered through a 0.22 µm spin filter (Agilent technologies, UK) for 2 min at 16,000 x g. For each run, 120 µL of diluted serum sample were injected into the Agilent Human 14 MARS Column (4.6X100 mm) coupled with 1200 Series HPLC (Agilent technologies, UK). Sample depletion was carried out using the following 48 min isocratic elution: 100% buffer A for 20 min at 0.125 mL/min then 2.5 min at 0.7 mL/min followed by 10 min of 100% buffer B (Agilent technologies) at 0.7 mL/min and 15.5 min of

buffer A for the column equilibration. The flow-through was collected between 12 and 21 min with buffer A and the bound proteins were eluted between 26 and 30 min with buffer B. Each sample was injected several times in order to get sufficient protein quantity for proteomics analysis.

TCA/DOC precipitation and quantitation| Pooled top 14 depleted samples were further concentrated and desalted by TCA/DOC precipitation. Sodium deoxycholate (final concentration: 125 µg/mL) was added to the samples and the mix left for 15 min at RT before the addition of trichloroacetic acid (final concentration: 6 %). The samples were centrifuged for 10 min at 12,000 g. Ice cold acetone was added to the pellets and the samples were centrifuged at 12,000 g for 5 min at 4°C. The supernatants were discarded and the dried pellets were resuspended in 50 µL of buffer containing 6 M Urea and 100mM TrisHCl (pH 7.8). The Pierce BCA protein assay (Thermo Scientific, Basingstoke, UK) was used for protein quantitation.

SDS-PAGE and protein digestion| Equal amounts of proteins were separated onto a criterion XT Bis-Tris gel 4-12 % using XT MES running buffer (Biorad, UK). After the separation of the proteins, the gels were stained with Instant Blue (Expedeon Ltd, Harston, UK) for 10 min and transferred in distilled water for direct use.

For each gel, thirteen bands were cut into small gel pieces (1-2 mm3), transferred into a 1.5 mL tube and destained with 50 % methanol and 5 % acetic acid until color disappeared. Each band was reduced with 10 mM DTT for 30 min, alkylated with 50 mM iodoacetamide for 30 min and digested with trypsin at 5 ng/µL in 50 mM ammonium bicarbonate at 37°C overnight. Two incubations of 10 min with extraction buffer I (50 % acetonitrile, 5 % formic acid) and a third with extraction buffer II (85 % acetonitrile, 5 % formic acid) were used to extract the peptides from the gel pieces. Peptide mixtures were dried with vacuum centrifugation and frozen at -20°C until the MS/MS analysis.

Mass spectrometry analysis| LC-MS/MS of peptides was performed using a Q-TOF 6520 (Agilent Technologies) equipped with a nanoLC-chip cube. The HPLC consisted of a nanoflow analytical and a capillary loading pump (Agilent 1200 series). Peptides were enriched and separated via nano-LC (0.075 x 150 mm, packed with Zorbax 300SB-C18, 5 µm material, 300 Å pore size) integrated in the HPLC Chip (G4240-62001). For each mass spectrometry experiment, peptides were loaded onto the enrichment column with 100%

solvent A (2% acetonitrile with 0.1% formic acid). A two-step gradient generated at a flow rate of 0.6 μl/min was used for peptide elution. This included a linear gradient from 5% to 40% buffer B (95% acetonitrile with 0.1% formic acid) over 45 min followed by a sharp increase to 100% B within 10 min. The total run time, including column reconditioning, was 60 min. Full scan was acquired over a range of m/z 400-1700 at 5 spectra per second rate, and MS/MS over m/z 50-1700 at two spectra per second rate selecting the six most abundant doubly or triply charged precursor ions per cycle. Auto-MS/MS was performed with a total cycle time of 3.3 seconds. Selected precursor masses were excluded for 0.9 minutes in order to avoid repeated sequencing. Spectra were deconvoluted and analysed using Mass Hunter (v4.0) and Spectrum Mill software (Agilent).

MS/MS spectra generated above were extracted from the raw data in mzXML file format using Agilent trapper (version 4.3.0 Aug 2009) as conversion tool. The mzXML data were submitted to the Central Proteomics Facilities Pipeline (CPFP_OXFORD, version 1.3.0) search engine for protein identification.

Database searching and label-free protein quantitation| The identification and quantification of proteins was done using CPFP, which combines data from three search engines (Mascot, OMSSA and X! tandem k-score). Trypsin was the enzyme used for the digestion of the proteins and only one missed cleavage was allowed. The accepted tolerance for the precursor was 50 ppm and 0.1 Da for the fragment. The search encompassed 1+, 2+ and 3+ charge state, fixed modification for cysteine carbamidomethyl and variable modification for asparagine and glutamine deamidation, and methionine oxidation. The label-free analysis was carried out using the normalized spectral index SINQ and LC-MS Progenesis software (version 3.1.4003.30577).

References

1. Jallow M, Casals-Pascual C, Ackerman H, et al. Clinical features of severe malaria associated with death: a 13-year observational study in the Gambia. PLoS One **2012**; 7:e45645.
2. Severe falciparum malaria. World Health Organization, Communicable Diseases Cluster. Trans R Soc Trop Med Hyg **2000**; 94 Suppl 1:S1-90.
3. WorldHealthOrganization. Management of the child with serious infection or severe malnutrition. Guidelines for care at the first-referral level in developing countries. Geneva, **2000**.
4. Cherian T, Mulholland EK, Carlin JB, et al. Standardized interpretation of paediatric chest radiographs for the diagnosis of pneumonia in epidemiological studies. Bull World Health Organ **2005**; 83:353-9.

5. WorldHealthOrganizationPneumoniaVaccineTrialInvestigatorsGroup. Standardization of interpretation of chest radiographs for the diagnosis of pneumonia in children. Geneva: World Health Organization, Department of Vaccines and Biologicals, **2001**.
6. Adegbola RA, Falade AG, Sam BE, et al. The etiology of pneumonia in malnourished and well-nourished Gambian children. Pediatr Infect Dis J **1994**; 13:975-82.
7. Berkley JA, Lowe BS, Mwangi I, et al. Bacteremia among children admitted to a rural hospital in Kenya. N Engl J Med **2005**; 352:39-47.
8. Brent AJ, Ahmed I, Ndiritu M, et al. Incidence of clinically significant bacteraemia in children who present to hospital in Kenya: community-based observational study. Lancet **2006**; 367:482-8.
9. Trudgian DC, Ridlova G, Fischer R, et al. Comparative evaluation of label-free SINQ normalized spectral index quantitation in the central proteomics facilities pipeline. Proteomics **2010**; 11:2790-7.
10. Trudgian DC, Thomas B, McGowan SJ, Kessler BM, Salek M, Acuto O. CPFP: a central proteomics facilities pipeline. Bioinformatics; 26:1131-2.

Figures

Supplementary Figure 1| Proteomic Study workflow. Plasma samples from Gambian children were randomly selected and used for shotgun proteomic studies. Samples were separated in 3 different groups of 100 children according to disease severity. Individual samples (5µl of plasma) were pooled into 3 different groups (~165µl of plasma per batch) in each disease category and depleted of the top-14 most abundant plasma proteins (albumin, IgG, antitrypsin, IgA, transferrin, haptoglobin, fibrinogen, alpha2-macroglobulin, alpha1-acid glycoprotein, IgM, apolipoprotein AI, apolipoprotein AII, complement C3, and transthyretin). After protein precipitation and enzymatic digestion with trypsin, samples were analysed in an LC-MS/MS (Agilent Q-TOF 6520) and searched against the human proteome with a false-discovery rate of 1% calculated from target-decoy hits and relative (label-free) quantification was based on normalized spectral counts (SINQ). Proteins that were up-or down-regulated following a disease progression pattern (SP>NSP>C or vice versa) in two batches or more were selected as candidate biomarkers. Final selection for enzyme immuno assay (EIA) validation was based on these criteria and clinical and biological relevance.

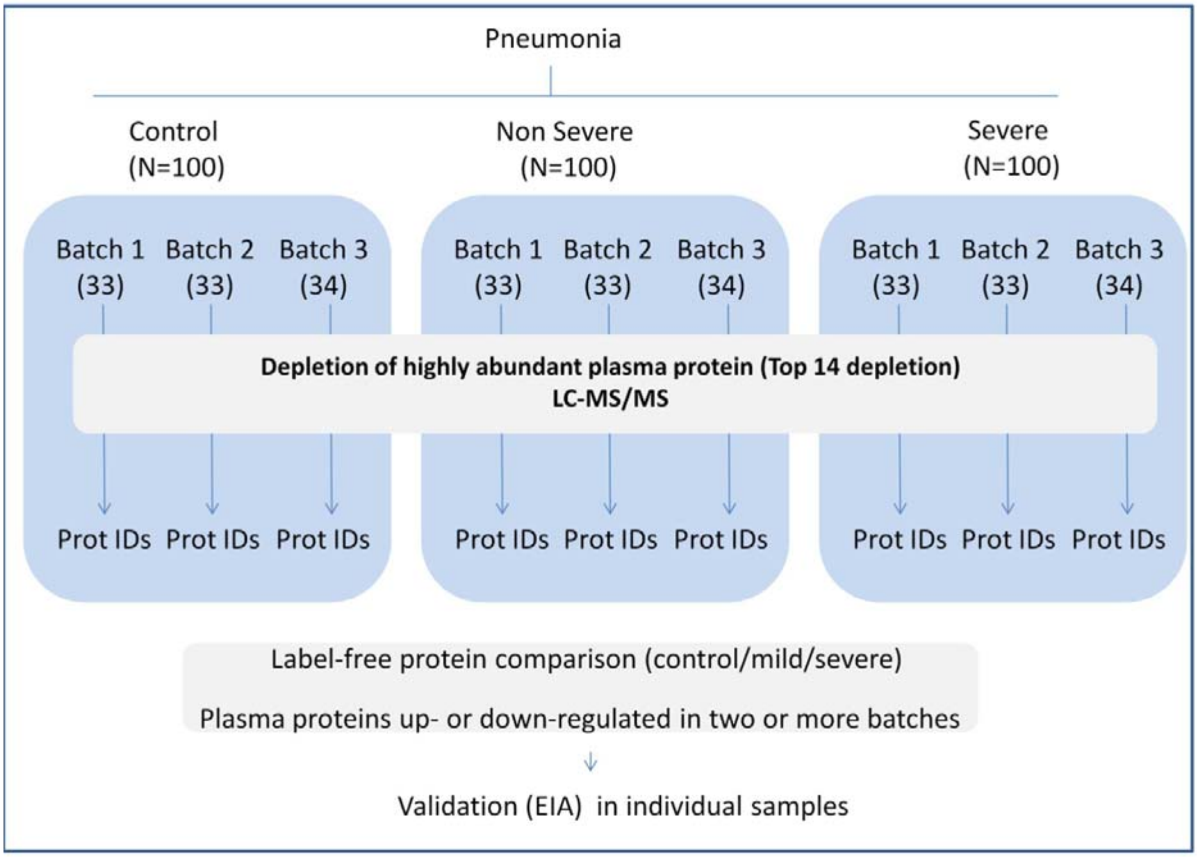

Supplementary Figure 2| Relative abundance of plasma proteins identified and quantified by liquid chromatography tandem-mass spectrometry in children with severe pneumonia (SP), non-severe pneumonia (NSP) and controls (Ctrl). Protein abundance was quantified using normalized spectral counts quantitation (SINQ). Error bars indicate standard error of the mean of three independent pools of samples. Red and green bars denote up- and –down-regulation, respectively. Arrows indicate candidate markers of disease progression.

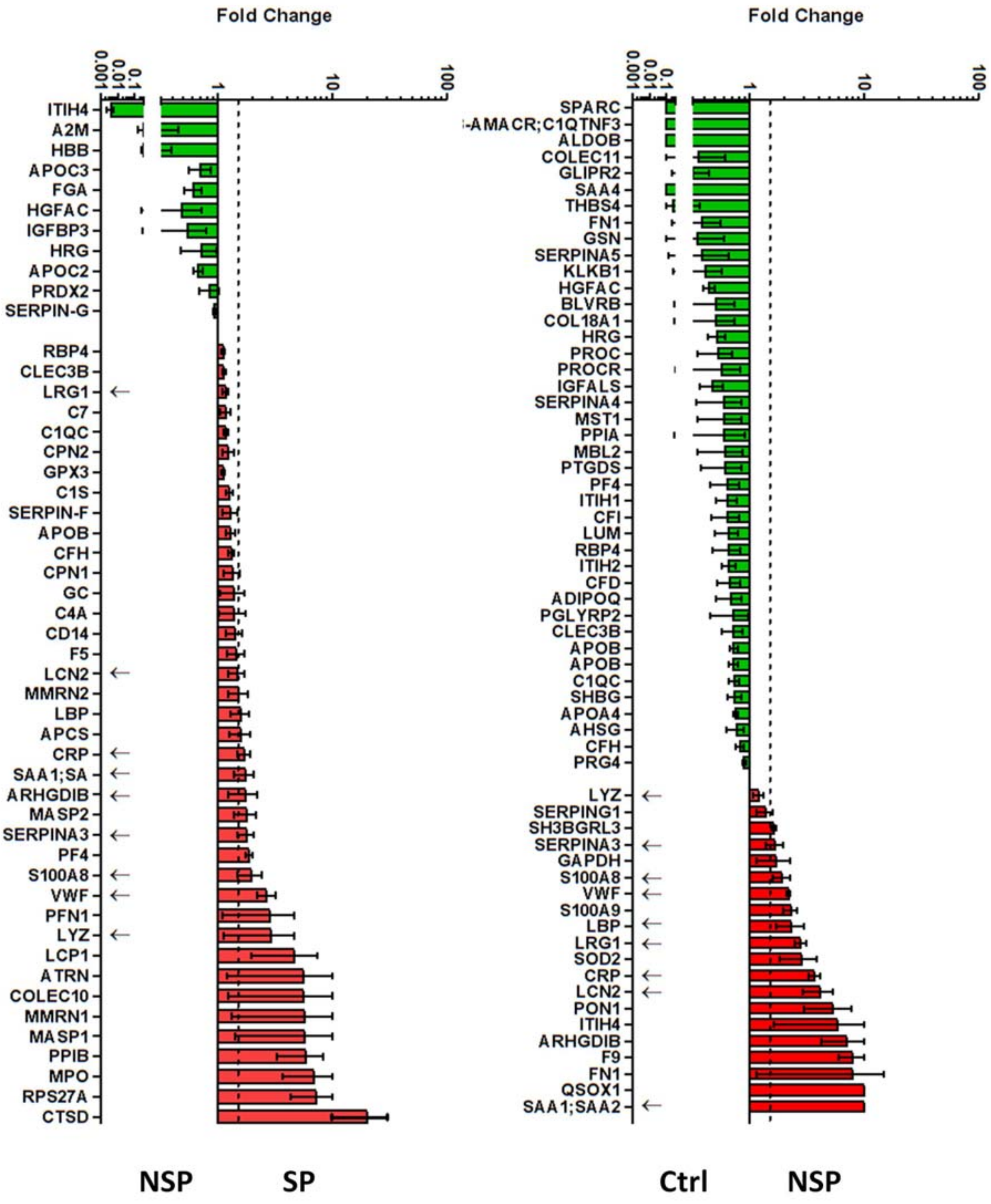

Supplementary Figure 3| Validation of candidate biomarkers in individual plasma samples by ELISA in clinical groups with different severity. Bars indicate median concentration and error bars denote interquartile range. NSP: non-severe pneumonia, SP: severe pneumonia, VSP: very severe pneumonia. Differences across groups was highly significant for all 3 biomarkers (Kruskal-Wallis, P<0.0001)

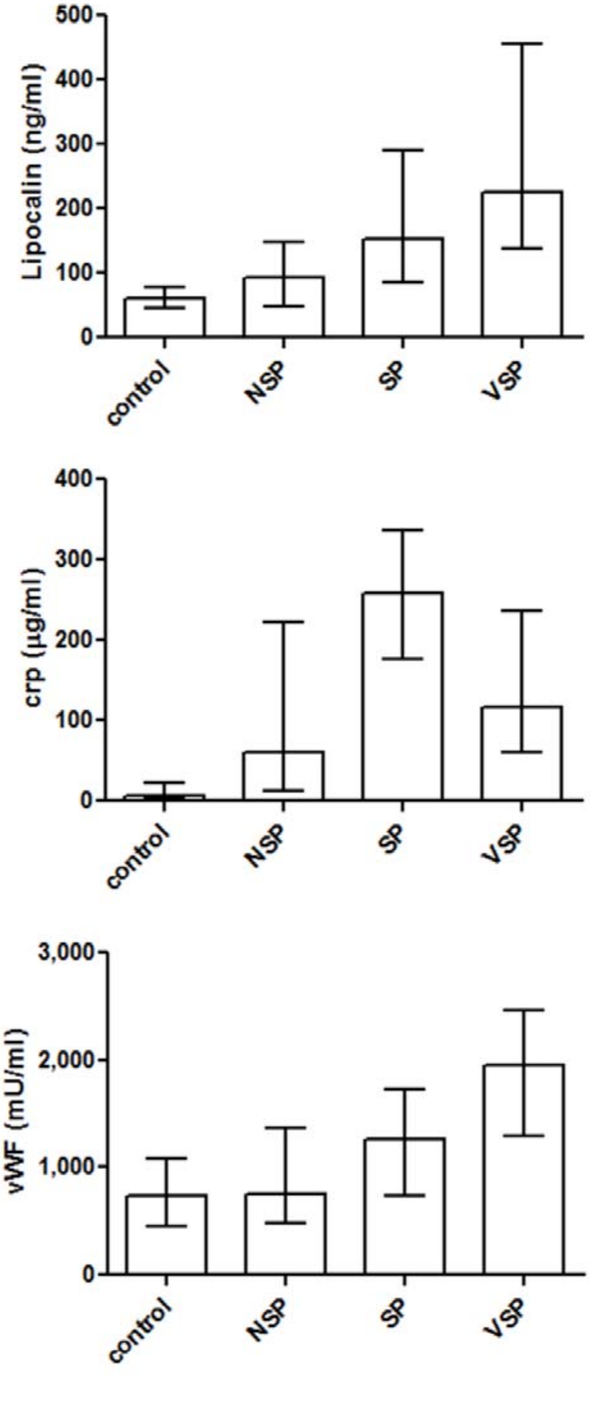

Supplementary Figure 4| Diagnostic performance of the best model to predict severe pneumonia stratified by seasonality. ROC curves show differences in sensitivity and

specificity of the combination of clinical features (respiratory rate and crepitations) and molecular markers (Lpc-2 and CRP) to predict severe pneumonia (versus mild pneumonia). The analysis shows ROC analysis stratified by season of enrolment in the study, 65 patients enrolled in the dry season versus 85 patients in the rainy season. The proportion of severe and mild pneumonia cases did not vary significantly with season (chi-square: 0.46). Blue circles denote rainy/malaria season (June to November) and empty circles denote dry season.

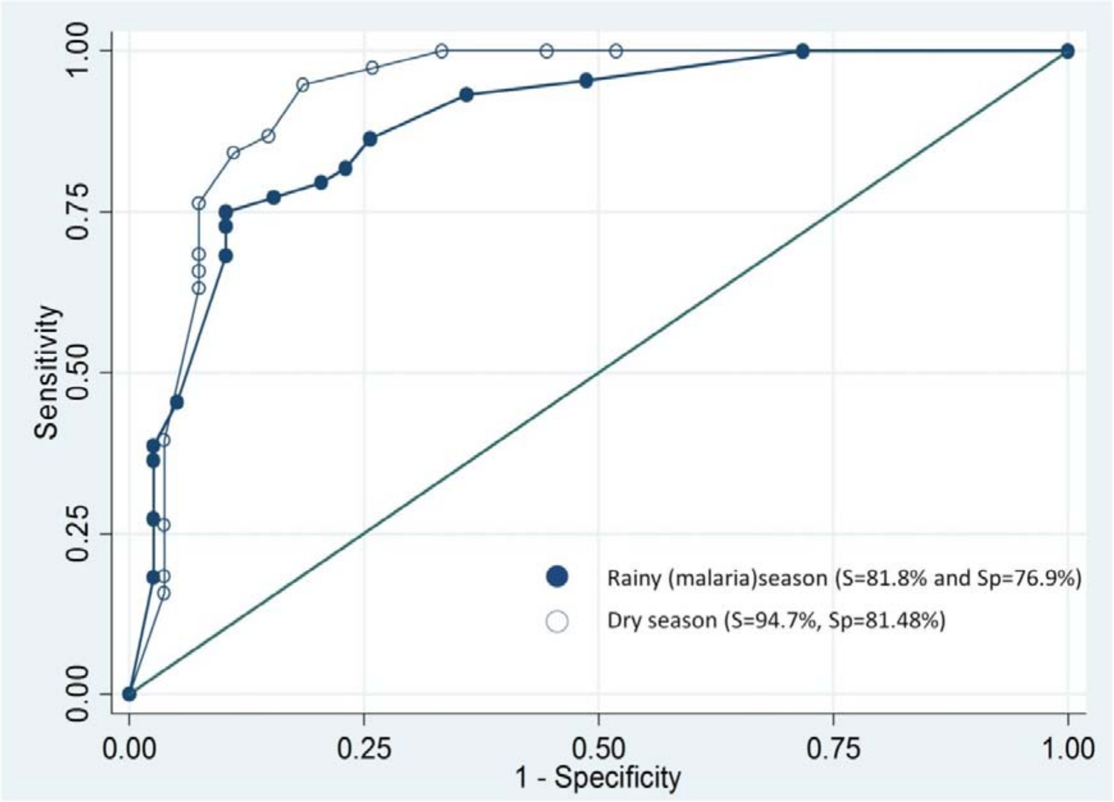

Supplementary Figure 5| Diagnostic performance of the best model to predict severe pneumonia using the new definition of severe pneumonia (WHO, 2013). Using the 2013 criteria, 15 cases previously allocated to the severe pneumonia group (out of 108) were now allocated to the NSP group. Lpc-2: lipocalin-2, CRP: C-reactive protein, SP: severe pneumonia, NSP: non-severe pneumonia.

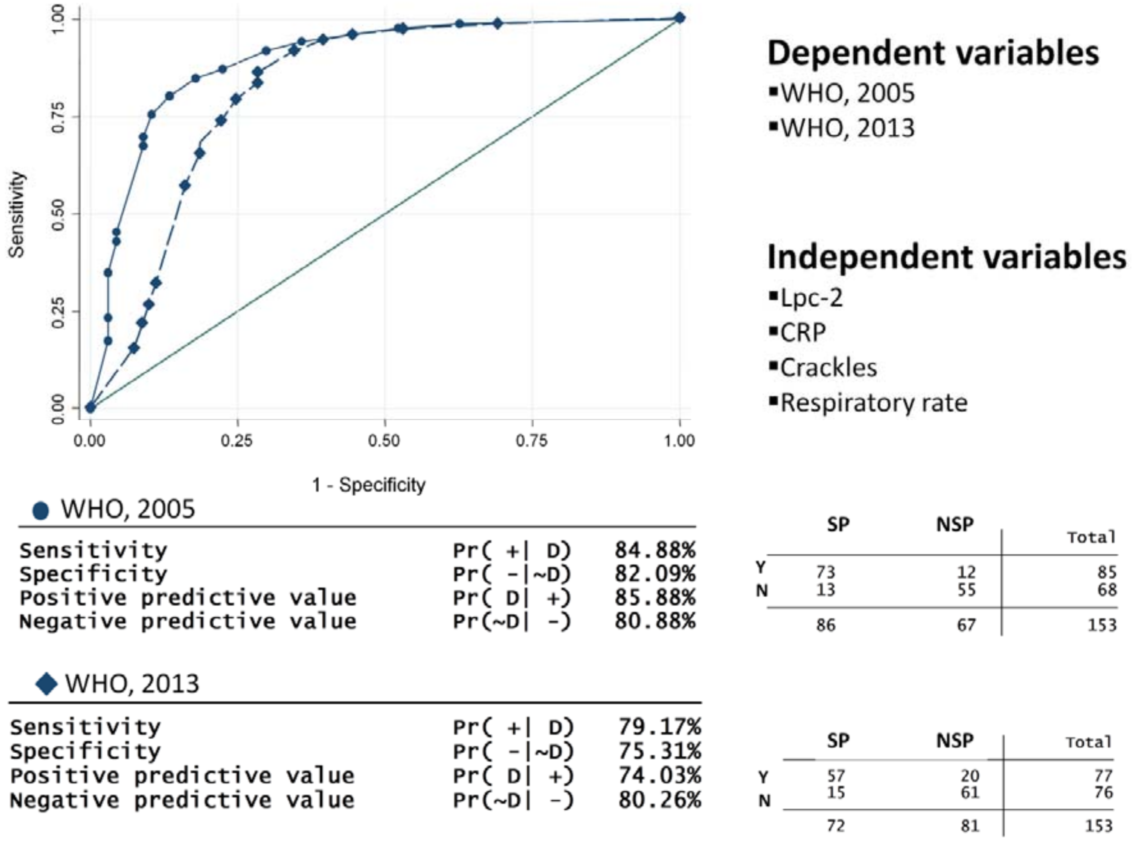

● WHO, 2005

Sensitivity	Pr(+| D)	84.88%
Specificity	Pr(-|~D)	82.09%
Positive predictive value	Pr(D| +)	85.88%
Negative predictive value	Pr(~D| -)	80.88%

	SP	NSP	Total
Y	73	12	85
N	13	55	68
	86	67	153

◆ WHO, 2013

Sensitivity	Pr(+| D)	79.17%
Specificity	Pr(-|~D)	75.31%
Positive predictive value	Pr(D| +)	74.03%
Negative predictive value	Pr(~D| -)	80.26%

	SP	NSP	Total
Y	57	20	77
N	15	61	76
	72	81	153

Supplementary Figure 6| Diagnostic performance of the best model to predict severe pneumonia using the new definition of severe pneumonia (WHO, 2013) compared with radiological changes (CXR, end-point consolidation), blood culture positivity and presence of crackles. Lpc-2: lipocalin-2, CRP: C-reactive protein, RR: respiratory rate, PPV: positive predictive value, NPV: negative predictive value; SP: severe pneumonia, NSP: non-severe pneumonia.

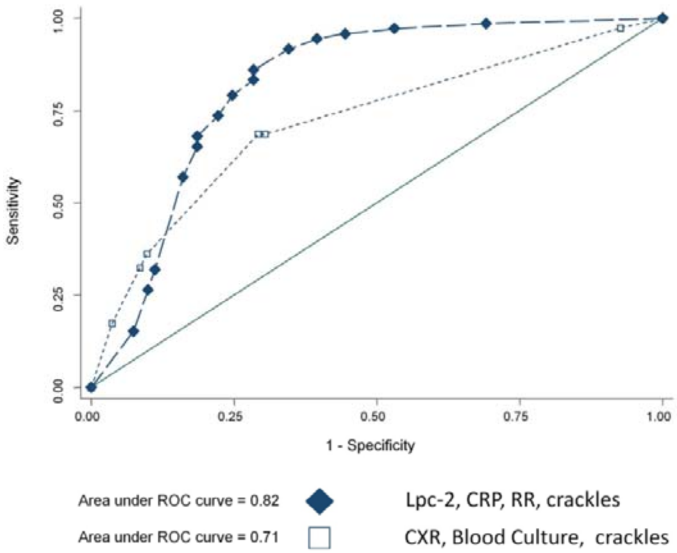

Clinical feature	Sensitivity	Specificity	PPV	NPV
	%	%	%	%
End point consolidation	37.3	90.2	79.48	58.7
Crackles	51.1	80.5	65.6	69.3
Positive blood culture	6.17	91.1	33.3	57.5
**Combined**	**68.7**	**70.7**	**69.6**	**69.8**

End point consolidation

	NSP	SP	Total
Y	74	52	126
N	8	31	39
	82	83	165

Blood culture

	NSP	SP	Total
Y	103	76	179
N	10	5	15
	113	81	194

Crackles

	NSP	SP	Total
Y	95	42	137
N	23	44	67
	118	86	204

Supplementary Figure 7| Plasma haptoglobin concentration in Gambian children. Data show mean values and standard error of the mean. NSP: non-severe pneumonia, SP: severe pneumonia, SPH: severe pneumonia with hypoxemia (SaO_2<90), SM: severe malaria with respiratory distress.

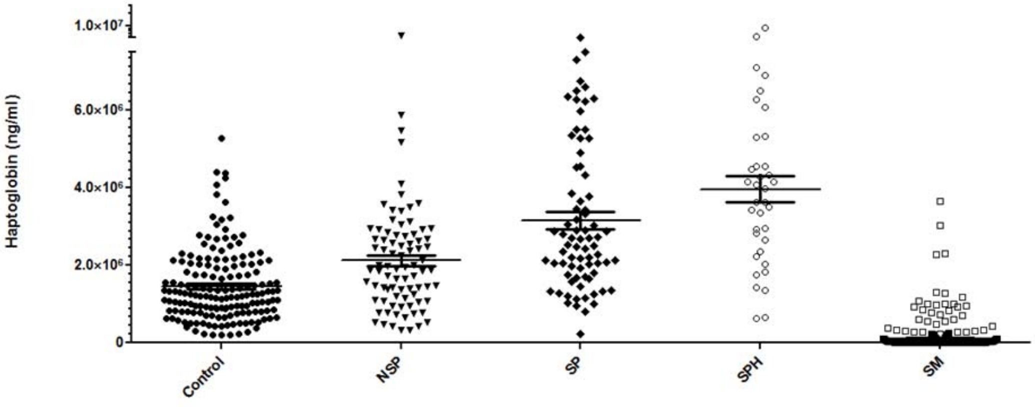

Supplementary Figure 8| Haptoglobin concentration in Kenyan children. Histogram shows the distribution of plasma haptoglobin concentration in Kenyan children by clinical phenotype (a). Scatter plot of haptoglobin and Lpc-2 values for those children who were admitted with SM (b) and both pneumonia and severe malaria (c). Figures indicate $\log_{10}$ *P.falciparum* density. * P<0.05

(a)

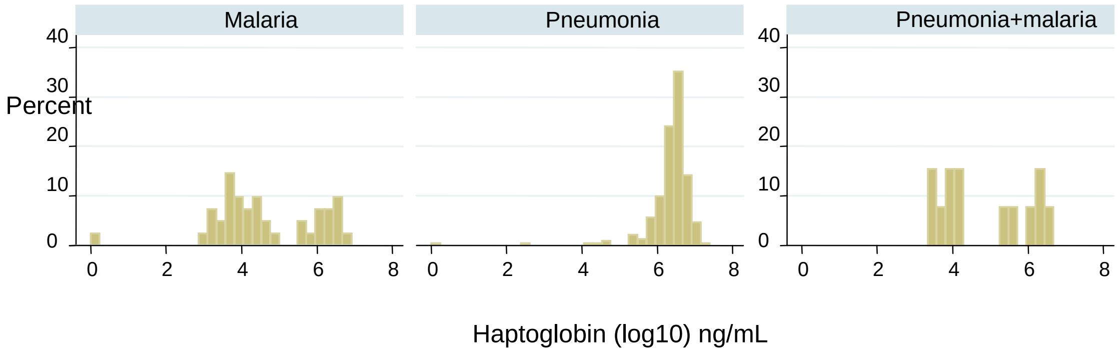

(b)

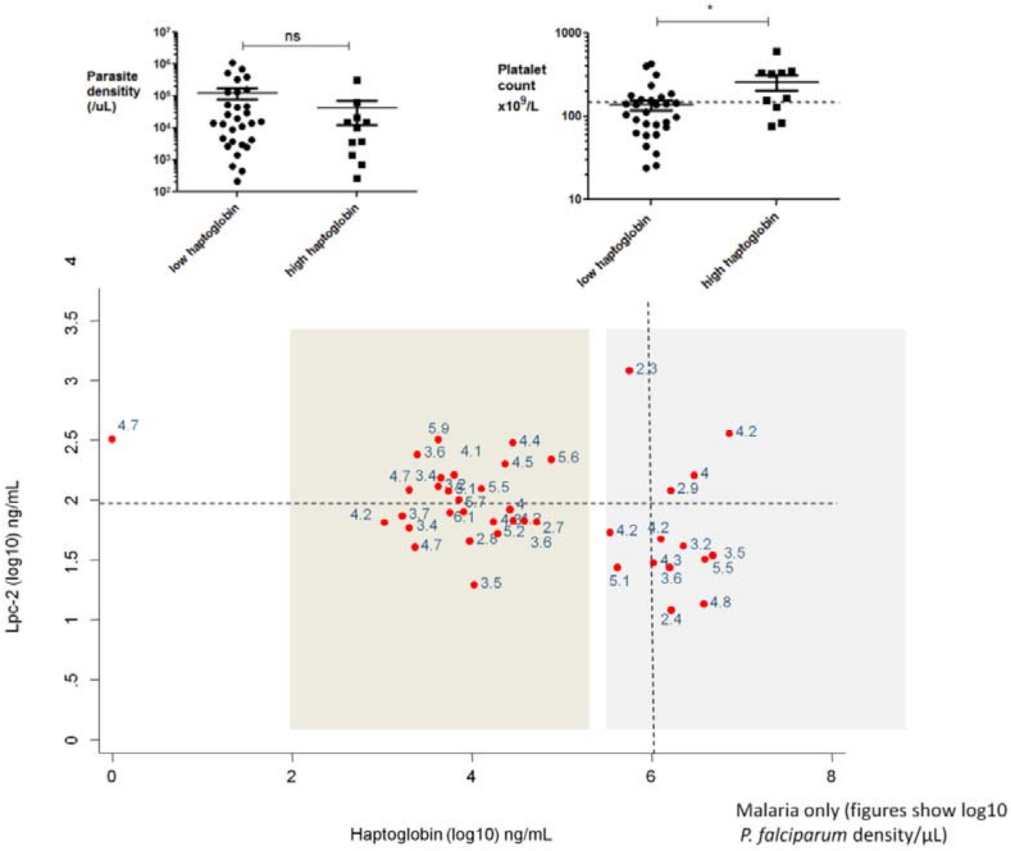

(c)

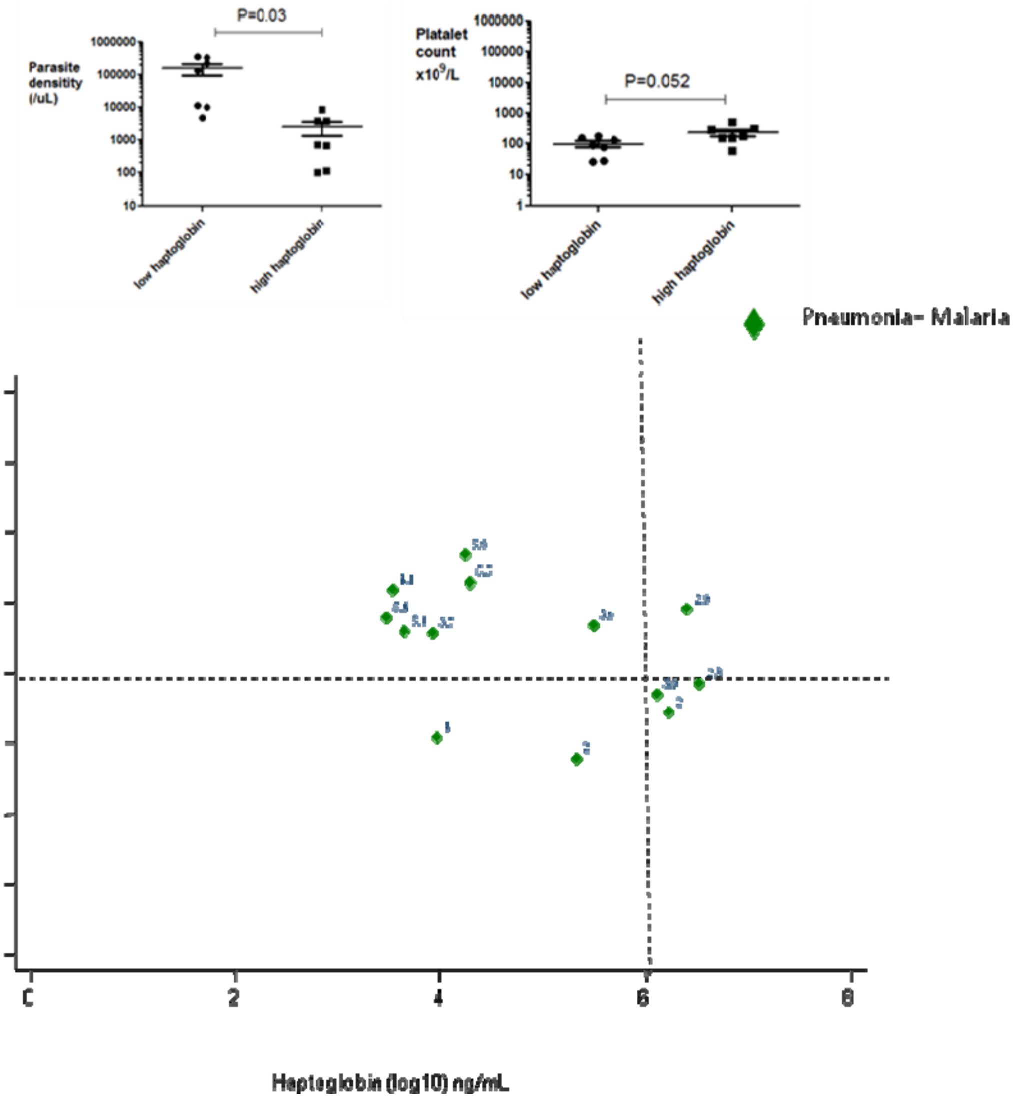
P=0.03
Parasite density (/uL)
1000000
100000
10000
1000
100
10
low haptoglobin
high haptoglobin
Platalet count x10⁹/L
P=0.052
1000000
100000
10000
1000
100
10
1
low haptoglobin
high haptoglobin
Pneumonia- Malaria
Lpc-2 (log10) ng/mL
4
3.5
3
2.5
2
1.5
1
.5
0
0
2
4
6
8
Heptoglobin (log10) ng/mL

Supplementary Figure 9|Diagnostic performance of Lpc-2 and haptoglobin to predict bacteremia (a) and to distinguish respiratory distress caused by malaria or severe pneumonia (b) stratified by age (median age=13.9 months).

(a)

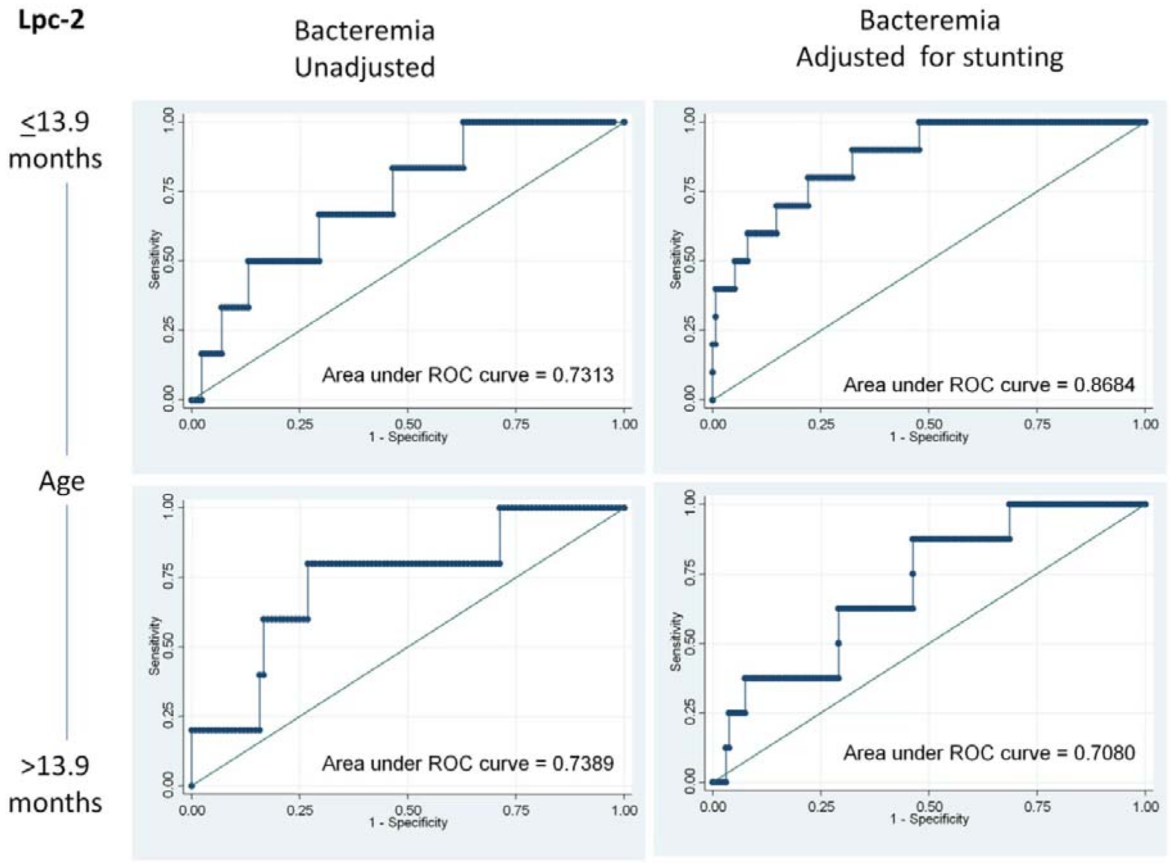

(b)

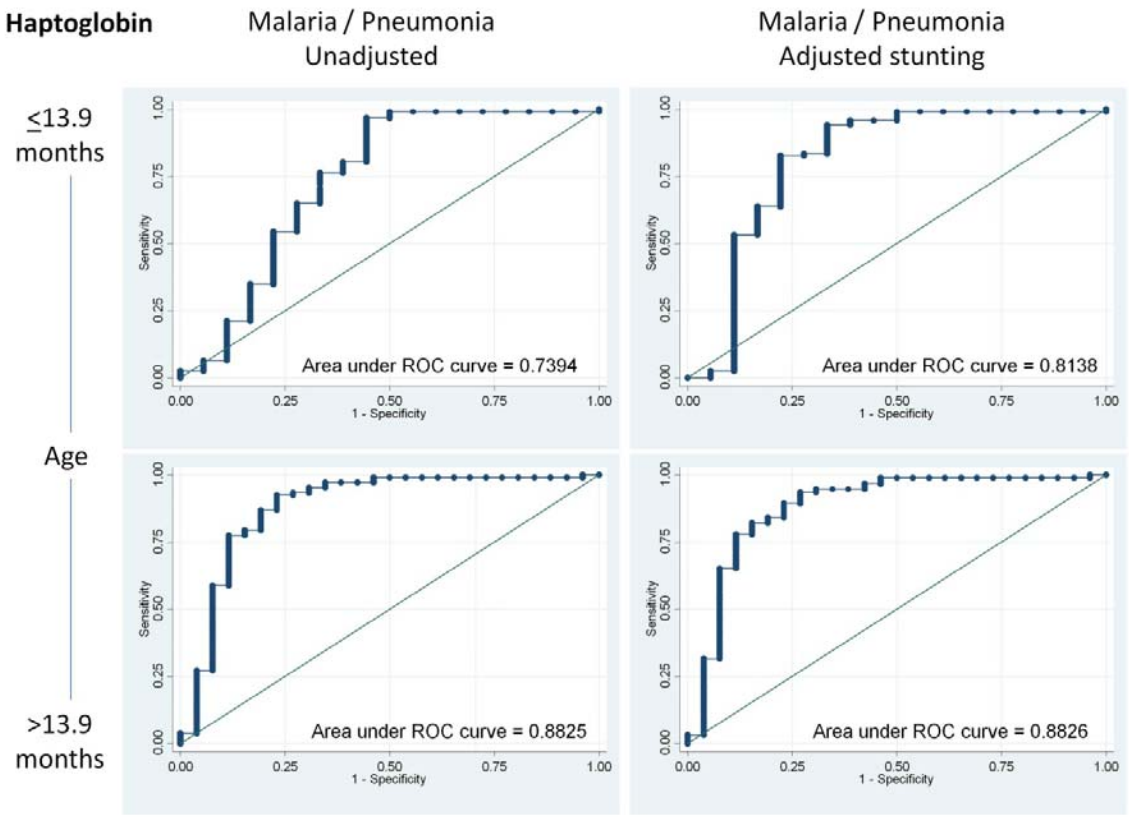
Haptoglobin
Malaria / Pneumonia
Unadjusted
Malaria / Pneumonia
Adjusted stunting
≤13.9 months
Age
>13.9 months
Sensitivity
1 - Specificity
Area under ROC curve = 0.7394
Area under ROC curve = 0.8138
Area under ROC curve = 0.8825
Area under ROC curve = 0.8826

Supplementary Figure 10| Lpc-2 and bloodstream infection in Kenyan children. Data show histograms describing the nutritional status (height-for-age, HAZ) in the population studied in Kenyan and Gambian children (a). ROC curves compare the diagnostic performance of Lpc-2 to predict bacteremia in Kenyan children with and without statistical adjustment for HAZ and presence of oral candidiasis (b). Multivariate logistic regression model using bacteremia as dependent variables and Lpc-2 concentration, HAZ and oral candidiasis as independent variables (c). Adjusted AUROC were generated using the 'lroc' function (Stata, 11) from the adjusted logistic regression model.

(a)

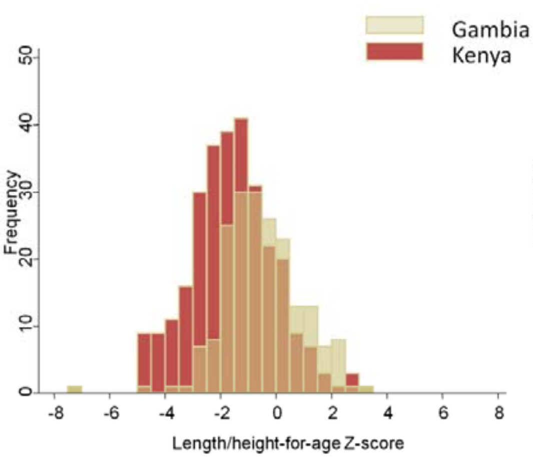

(b)

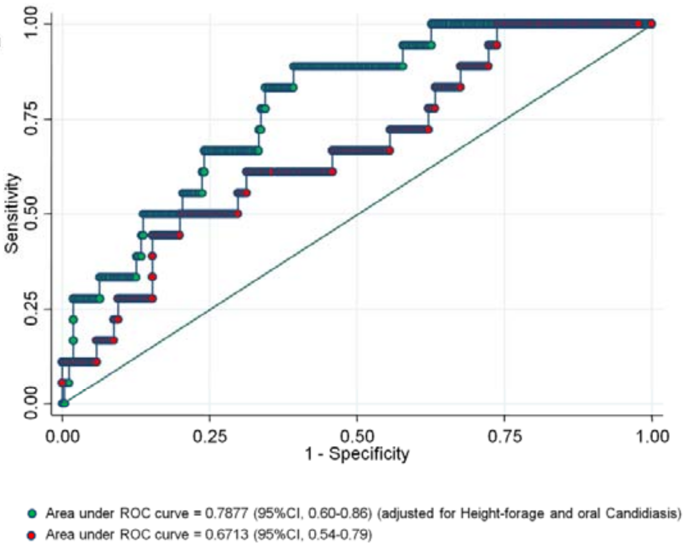

(c)

Positive blood culture	Odds Ratio	Std. Err.	z	P>|z|	[95% Conf.interval]	
lpc-2 (log10) ng/mL	5.12	3.30	2.53	0.011	1.45	18.13
Height-for-age	0.65	0.12	-2.33	0.02	0.45	0.93
Presence of oral candidiasis	4.39	3.30	1.96	0.05	1.00	19.19

Supplementary Figure 11. Lipocalin-2 is associated with *Streptococcus pneumoniae* - positive blood cultures. Error bars indicate standard deviation.

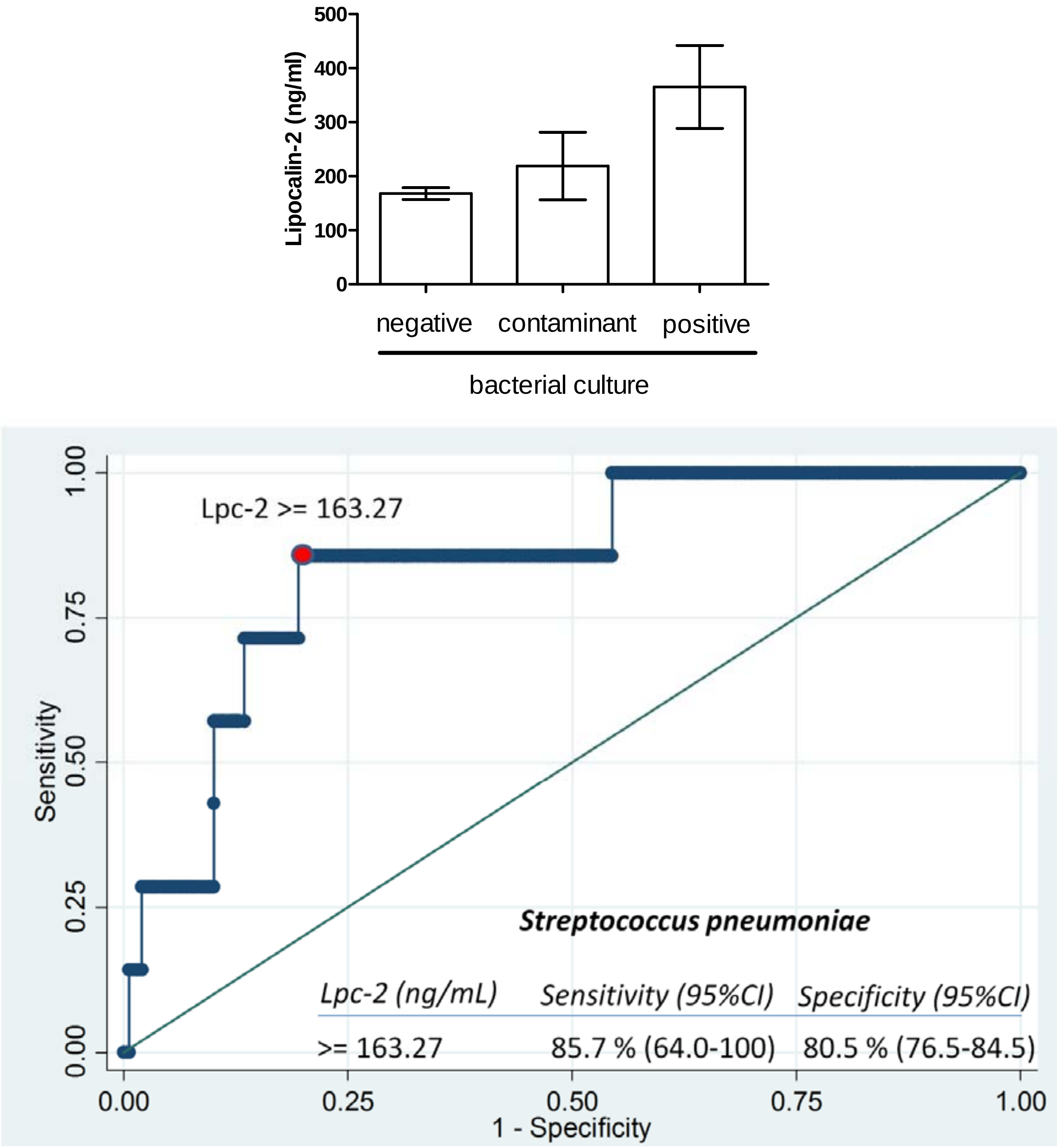

Tables

Supplementary Table 1| Clinical study sites and patients enrolled in the discovery and validation studies.

**Discovery**		Gambian Severe Pneumonia Study (Banjul)			
	Total study numbers	Community Controls N=801	Non-severe N=322	Severe N=458	Very severe N=36
	Randomly selected for proteomic study	N=100	N=100	N=100	
				$SaO_2 \geq 90\%$ N=91	$SaO_2 < 90\%$ Very severe cases N=9 (Banjul)
					Additional very severe cases N=25 (Basse)
					Total Very Severe cases N=34
**Validation 1: PNEUMONIA**					
	Number with clinical data available	N=186	N=96	N=76	N=32
	Number with samples available	N=186	N=96	N=76	N=32
	Total available for EIA analysis	N=160	N=77	N=73	N=32
**Validation 1: SEVERE MALARIA**		Gambian Severe Malaria Study (N=2,915)			
	Number with samples available for EIA validation	N=301			
**Validation 2: PNEUMONIA+MALARIA**		Kenyan Respiratory Distress study (N=293)			
		Malaria with Respiratory distress	Pneumonia	Pneumonia and Malaria	
	Total study numbers with clinical data and samples available for EIA validation	N=41	N=238	N=14	

EIA: Enzyme Immunoassay

Supplementary Table 2| Identification of differentially regulated proteins in patients with severe pneumonia compared with non-severe pneumonia.

**Prot ID**	Gene Name	**Protein Name**
IPI00478003	A2M	alpha-2-macroglobulin
IPI00022391	APCS	amyloid P component, serum
IPI01014270	APOB	apolipoprotein B (including Ag(x) antigen)
IPI00021856	APOC2	apolipoprotein C-II
IPI00021857	APOC3	apolipoprotein C-III
IPI00003817	ARHGDIB	Rho GDP dissociation inhibitor (GDI) beta
IPI00162735	ATRN	attractin
IPI00022394	C1QC	complement component 1, q subcomponent, C chain
IPI00017696	C1S	complement component 1, s subcomponent
IPI00643525	C4B	complement component 4B (Chido blood group)
IPI00909594	C7	complement component 7
IPI00029260	CD14	CD14 molecule
IPI00029739	CFH	complement factor H
IPI00009028	CLEC3B	C-type lectin domain family 3, member B
IPI00007917	COLEC10	collectin sub-family member 10 (C-type lectin)
IPI00010295	CPN1	carboxypeptidase N, polypeptide 1
IPI00479116	CPN2	carboxypeptidase N, polypeptide 2
IPI00022389	CRP	C-reactive protein
IPI00011218	CSF1R	colony stimulating factor 1 receptor
IPI00011229	CTSD	cathepsin D
IPI00022937	F5	coagulation factor V (proaccelerin, labile factor)
IPI00021885	FGA	fibrinogen alpha chain
IPI00555812	GC	group-specific component (vitamin D binding protein)
IPI00026199	GPX3	glutathione peroxidase 3 (plasma)
IPI00654755	HBB	hemoglobin, beta
IPI00029193	HGFAC	HGF activator
IPI00022371	HRG	histidine-rich glycoprotein
IPI00024284	HSPG2	heparan sulfate proteoglycan 2
IPI00018305	IGFBP3	insulin-like growth factor binding protein 3
IPI00944960	ITIH4	inter-alpha (globulin) inhibitor H4
IPI00032311	LBP	lipopolysaccharide binding protein
IPI00299547	LCN2	lipocalin 2
IPI00010471	LCP1	lymphocyte cytosolic protein 1 (L-plastin)
IPI00022417	LRG1	leucine-rich alpha-2-glycoprotein 1
IPI00019038	LYZ	lysozyme
IPI00290283	MASP1	mannan-binding lectin serine peptidase 1
IPI00294713	MASP2	mannan-binding lectin serine peptidase 2
IPI00012269	MMRN1	multimerin 1
IPI00015525	MMRN2	multimerin 2
IPI00007244	MPO	myeloperoxidase
IPI00022446	PF4	platelet factor 4
IPI00216691	PFN1	profilin 1
IPI00646304	PPIB	peptidylprolyl isomerase B (cyclophilin B)
IPI00027350	PRDX2	peroxiredoxin 2
IPI00022420	RBP4	retinol binding protein 4, plasma
IPI00179330	RPS27A	ribosomal protein S27a
IPI00007047	S100A8	S100 calcium binding protein A8
IPI00550991	SERPINA3	serpin peptidase inhibitor, (alpha-1 antiproteinase, antitrypsin),
IPI00006114	SERPINF1	serpin peptidase inhibitor, (alpha-2 antiplasmin)
IPI00291866	SERPING1	serpin peptidase inhibitor, clade G (C1 inhibitor), member 1
IPI00744692	TALDO1	transaldolase 1
IPI00023014	VWF	von Willebrand factor

Supplementary Table 3| Identification of differentially regulated proteins in patients with non-severe pneumonia compared with controls.

Prot ID	Gene	Protein ID
**IPI00020019**	ADIPOQ	adiponectin, C1Q and collagen domain containing
**IPI00022431**	AHSG	alpha-2-HS-glycoprotein
**IPI00218407**	ALDOB	aldolase B, fructose-bisphosphate
**IPI00304273**	APOA4	apolipoprotein A-IV
**IPI01014270**	APOB	apolipoprotein B (including Ag(x) antigen)
**IPI00299435**	APOF	apolipoprotein F
**IPI00003817**	ARHGDIB	Rho GDP dissociation inhibitor (GDI) beta
**IPI00783862**	BLVRB	biliverdin reductase B (flavin reductase (NADPH))
**IPI00022394**	C1QC	complement component 1, q subcomponent, C chain
**IPI00165972**	CFD	complement factor D (adipsin)
**IPI00029739**	CFH	complement factor H
**IPI01012711**	CFI	complement factor I
**IPI00009028**	CLEC3B	C-type lectin domain family 3, member B
**IPI00022822**	COL18A1	collagen, type XVIII, alpha 1
**IPI00031490**	COLEC11	collectin sub-family member 11
**IPI00022389**	CRP	C-reactive protein, pentraxin-related
**IPI00296176**	F9	coagulation factor IX
**IPI00022418**	FN1	fibronectin 1
**IPI00922213**	FN1	fibronectin 1
**IPI00219018**	GAPDH	glyceraldehyde-3-phosphate dehydrogenase
**IPI00007067**	GLIPR2	GLI pathogenesis-related 2
**IPI00026314**	GSN	gelsolin
**IPI00029193**	HGFAC	HGF activator
**IPI00022371**	HRG	histidine-rich glycoprotein
**IPI00020996**	IGFALS	insulin-like growth factor binding protein, acid labile subunit
**IPI01014157**	ITIH1	inter-alpha (globulin) inhibitor H1
**IPI00305461**	ITIH2	inter-alpha (globulin) inhibitor H2
**IPI00944960**	ITIH4	inter-alpha (globulin) inhibitor H4
**IPI00654888**	KLKB1	kallikrein B, plasma (Fletcher factor) 1
**IPI00032311**	LBP	lipopolysaccharide binding protein
**IPI00299547**	LCN2	lipocalin 2
**IPI00023673**	LGALS3BP	lectin, galactoside-binding, soluble, 3 binding protein
**IPI00022417**	LRG1	leucine-rich alpha-2-glycoprotein 1
**IPI00020986**	LUM	lumican
**IPI00019038**	LYZ	lysozyme
**IPI00004373**	MBL2	mannose-binding lectin (protein C) 2, soluble
**IPI00925540**	MST1	macrophage stimulating 1 (hepatocyte growth factor-like)
**IPI00022446**	PF4	platelet factor 4
**IPI00163207**	PGLYRP2	peptidoglycan recognition protein 2
**IPI00218732**	PON1	paraoxonase 1
**IPI00419585**	PPIA	peptidylprolyl isomerase A (cyclophilin A)
**IPI00024825**	PRG4	proteoglycan 4
**IPI00021817**	PROC	protein C (inactivator of coagulation factors Va and VIIIa)
**IPI00009276**	PROCR	protein C receptor, endothelial
**IPI00013179**	PTGDS	prostaglandin D2 synthase 21kDa (brain)
**IPI00003590**	QSOX1	quiescin Q6 sulfhydryl oxidase 1
**IPI00022420**	RBP4	retinol binding protein 4, plasma

**IPI00007047**	S100A8	S100 calcium binding protein A8
**IPI00027462**	S100A9	S100 calcium binding protein A9
**IPI00019399**	SAA4	serum amyloid A4, constitutive
**IPI00550991**	SERPINA3	serpin peptidase inhibitor, clade A (alpha-1 antiproteinase, antitrypsin), member 3
**IPI00328609**	SERPINA4	serpin peptidase inhibitor, clade A (alpha-1 antiproteinase, antitrypsin), member 4
**IPI00007221**	SERPINA5	serpin peptidase inhibitor, clade A (alpha-1 antiproteinase, antitrypsin), member 5
**IPI00291866**	SERPING1	serpin peptidase inhibitor, clade G (C1 inhibitor), member 1
**IPI00010402**	SH3BGRL3	SH3 domain binding glutamic acid-rich protein like 3
**IPI00023019**	SHBG	sex hormone-binding globulin
**IPI00022314**	SOD2	superoxide dismutase 2, mitochondrial
**IPI00014572**	SPARC	secreted protein, acidic, cysteine-rich (osteonectin)
**IPI00328550**	THBS4	thrombospondin 4
**IPI00023014**	VWF	von Willebrand factor

Supplementary Table 4. Diagnostic performance of selected features to predict severe pneumonia (data correspond to Figure 1).

Lpc-2		NSP	SP	Total	S	Sp	PPV	NPV
	<118	54	30	84				
	>=118	23	75	98	*71.4*	*70.1*	*76.5*	*64.2*
	Total	77	105	182				
**CRP**		**NSP**	**SP**	**Total**				
	<157	46	28	74				
	>=157	34	61	95	*68.5*	*57.5*	*64.2*	*68.2*
	Total	80	89	169				
**Respiratory rate**		**NSP**	**SP**	**Total**				
	<58	71	33	104	*69.4*	*73.9*	*75*	*68.2*
	>=58	26	75	100				
	Total	96	108	204				
**Crackles (stethoscope)**		**NSP**	**SP**	**Total**				
	YES	87	50	137	*53.7*	*90.6*	*86.5*	*63.5*
	NO	9	58	67				
	Total	96	108	204				

Supplementary Table 5. Diagnostic performance of LPc-2 to predict *S.pneumoniae* bacteremia (data correspond to Figure 2).

Lpc-2	*S pneumoniae*		Total
	N	Y	
<163	281	1	282
>=163	68	6	74
Total	349	7	356

S	Sp	PPV	NPV
85.7	*80.5*	*8.1*	*99.6*

Supplementary Table 6. Clinical features and bacteremia. The table describes a univariate logistic regression analysis using blood culture as the dependent variable and clinical features as independent variables**.** N: number of patients with data available, OR: odds ratio, 95%CI: 95% confidence interval.

Clinical features	N	OR	95% CI	*P* value
Temperature (°C)	194	1.63	0.92-2.86	0.08
Respiratory rate	194	1.01	0.96-1.05	0.69
Indrawing	194	1.1	0.38-3.18	0.85
Grunting	194	1.58	0.51-4.88	0.425
Nasal flaring	194	1.01	0.35-2.90	0.98
